# ILC3s mediate intestinal immune-epithelial interactions via TGF-β1 activation

**DOI:** 10.1016/j.mucimm.2025.11.013

**Published:** 2026-04

**Authors:** Diana Coman, John W. Bassett, Isabelle Coales, Ainize Peña-Cearra, Emily Read, Emma H. Kromann, Daniel Brice, Zuzanna Łukasik, Helena Paidassi, Matthew R. Hepworth, Robin J. Dart, Mark A. Travis, Jenny Mjösberg, Luke B. Roberts, Joana F. Neves

**Affiliations:** a Centre for Host-Microbiome Interactions, King’s College London, London, United Kingdom; b Center for Infectious Medicine, Department of Medicine Huddinge, Karolinska Institutet, Karolinska University Hospital Huddinge, Stockholm, Sweden; c Department of Immunology, Microbiology and Parasitology, Faculty of Medicine and Nursing, University of the Basque Country (EHU), Bilbao, Spain; d The Blizard Institute, Queen Mary University London, London, United Kingdom; e Lydia Becker Institute of Immunology and Inflammation, University of Manchester, Manchester, United Kingdom; f Wellcome Trust Centre for Cell-Matrix Research, University of Manchester, Manchester, United Kingdom; g Division of Immunology, Immunity to Infection and Respiratory Medicine, Faculty of Biology, Medicine and Health, Manchester Academic Health Sciences Centre, University of Manchester, Manchester, United Kingdom; h Department for Inflammation Biology, School of Immunology & Microbial Sciences, King’s College London, London, United Kingdom; i Department of Rheumatology, Faculty of Medicine and Health Sciences, Ghent University and Unit for Molecular Immunology and Inflammation, VIB-UGent Center for Inflammation Research, Ghent, Belgium; j Centre International de Recherche en Infectiologie (CIRI), Univ Lyon, Inserm, U1111 Université Claude Bernard Lyon 1, CNRS, ENS de Lyon, Lyon, France; k Immunosurveillance Laboratory, The Francis Crick Institute, London, United Kingdom; l Department of Gastroenterology, Guy’s and St Thomas’ Foundation Trust, London, United Kingdom; m Clinical Lung- and Allergy Research Unit, Department of Medicine Huddinge, Karolinska Institutet, Stockholm, Sweden; n The Department of Respiratory Medicine and Allergy, Karolinska University Hospital Huddinge, Stockholm, Sweden

**Keywords:** Innate Lymphoid Cells, ILC3, TGF-β1, Regulatory T cells, Organoids, Intestine

## Abstract

Inflammatory Bowel Disease (IBD) is a chronic inflammatory condition affecting the gastrointestinal tract. It is characterised by epithelial and immunological dysfunctions, including alterations in populations of Innate Lymphoid Cells (ILCs), including tissue-resident RORγt-expressing Group 3 ILCs (ILC3s). ILC3s contribute to the maintenance of intestinal homeostasis by closely interacting with both the epithelium and adaptive immune cells. Here, we reveal that murine and human ILC3s modulate these interactions through Transforming Growth Factor-Beta 1 (TGF-β1), a pleiotropic cytokine secreted in an inactive form. We show that ILC3s synthesise and activate latent TGF-β1 through mechanical and proteolytic pathways. ILC3s aid the induction of FoxP3^+^ regulatory T cells via TGF-β1 and promote a regenerative transcriptional signature in intestinal epithelial cells. The downstream impact of ILC3-derived TGF-β1 is conserved between mouse and humans, but the TGF-β1 activators expressed by ILC3 differ between the species. In IBD, where ILC3s are reduced in the inflamed intestinal regions, TGF-β1-production and activation machinery remains intact in ILC3s, suggesting this pathway is functional in disease and could be targeted to enhance intestinal homeostasis through promotion of epithelial regeneration and induction of regulatory T cells.

## Highlights

• Human and murine intestinal ILC3s are identified as critical producers of TGF-β1.
• ILC3s activate TGF-β1 via mechanical and proteolytic pathways.
• ILC3-derived TGF-β1 drives the induction of FoxP3^+^ T cells.
• ILC3s, via TGF-β1, upregulates genes associated with regeneration and epithelial to mesenchymal transition within intestinal epithelial cells.
• In IBD, the capacity for ILC3s to produce and activate TGF-β1 is maintained.

## Graphical abstract

**Figure:**
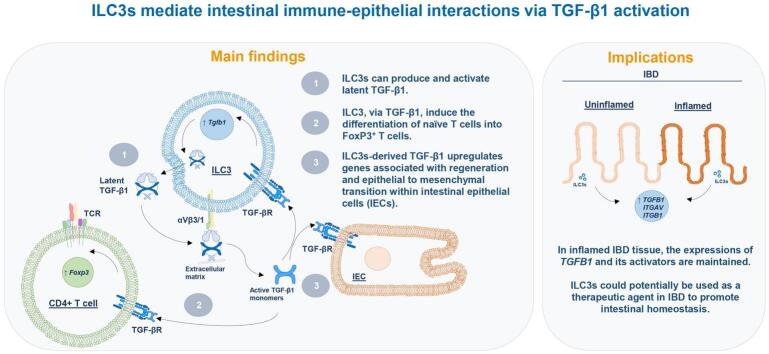

## Introduction

Inflammatory Bowel Disease (IBD), which encompasses Crohn’s disease (CD) and ulcerative colitis (UC), is a multifactorial disease that exhibits abnormal cellular interactions between the intestinal epithelium and local immune cells such as Innate Lymphoid Cells (ILCs). ILCs have been associated with both promotion of intestinal health, as well as contributing to inflammation in IBD. For instance, Group 1 ILCs (ILC1s), contribute to tissue damage through production of pro-inflammatory effectors such as IFNγ and TNF-α^1^, ^2^, ^3^. Conversely, RORγt^+^ Group 3 ILCs (ILC3s), through IL-22 and IL-17, modulate proliferation, differentiation and function of intestinal epithelial cells (IEC), thereby maintaining the integrity of the intestinal barrier^4^. Finally, ILC1s are increased at intestinal sites of active inflammation in IBD, while ILC3s show reduced abundance and altered functionality^5^ .

One route of action through which immune cells such as ILCs exert their effects is through the production of cytokines. In the context of intestinal homeostasis and disease, cytokines such as Transforming Growth Factor-Beta 1 (TGF-β1), are known to act as crucial mediators of crosstalk between IEC and other immunological compartments^6^, ^7^. For example, signalling via TGF-β1 is critical for the regulation of IECs, directly driving their regeneration and wound healing response^8^, ^9^, ^10^. Furthermore, TGF-β1 drives induction of regulatory T cells (Tregs) and is required for their suppression of effector T cell responses – processes which are dysregulated in IBD^11^. On the other hand, TGF-β1 can strongly contribute to aberrant pro-fibrotic processes associated with the chronic inflammatory environment observed in IBD^12^. Thus, it is critical to dissect the cellular and molecular mechanisms governing TGF-β1 to potentiate its beneficial functions in the gut.

TGF-β1 is secreted as a latent precursor complex and activated through dissociation of Latency Associated Peptide (LAP), leading to the release of the mature, bioactive TGF-β1 which can then interact with its signalling receptors^13^, ^14^. Bioactive TGF-β1 can act over short distances, between locally interacting populations^15^. Therefore, fully understanding how TGF-β1 performs its diverse roles requires detailed knowledge of the cells and mechanisms involved in its production, activation and responsiveness. Importantly, innate^16^, ^17^, ^18^, ^19^ and adaptive^20^, ^21^ leucocytes can modulate tissue bioavailability of TGF-β1. Recently, we identified ILC1 as producers of TGF-β1, which act to remodel IEC at sites of active inflammation during IBD^19^. Furthermore, recent studies show that RORγt-expressing antigen-presenting cells (APCs), such as ILC3s^22^, Janus^23^ and Thetis cells^24^ can express TGF-β1 activators on their surface, indicating a potential to activate TGF-β1. However, the capacity of ILC3s to produce, activate and regulate the function of TGF-β1 and the subsequent impact on immune and non-immune cells in intestinal health and IBD remains incompletely understood.

Here, we show that intestinal ILC3s both produce, and activate, latent TGF-β1, through mechanical and proteolytic pathways, in a manner that is further enhanced by exposure to TGF-β1. Using murine and human intestinal organoid models, we demonstrate that ILC3s modulate the phenotype and function of immune and epithelial cells in a TGF-β1-dependent manner. Through TGF-β1 signalling, ILC3s induce the expression of FoxP3 in naïve T cells, a hallmark of their differentiation to Tregs and promote a TGF-β1-signalling transcriptional signature on IECs. Importantly, we show that whilst this ILC3–TGF-β1 dependent pathway is conserved between mice and humans, the specific TGF-β1 activators differ. This underscores the critical need for human in vitro models to maximise translational potential of research into TGF-β1. Finally, we explore the potential implications of this pathway in IBD where we show that ILC3s retain the machinery to activate TGF-β1.

## Results

### Latent TGF-β1 is produced and activated by murine ILC3s

**Fig. 1:**
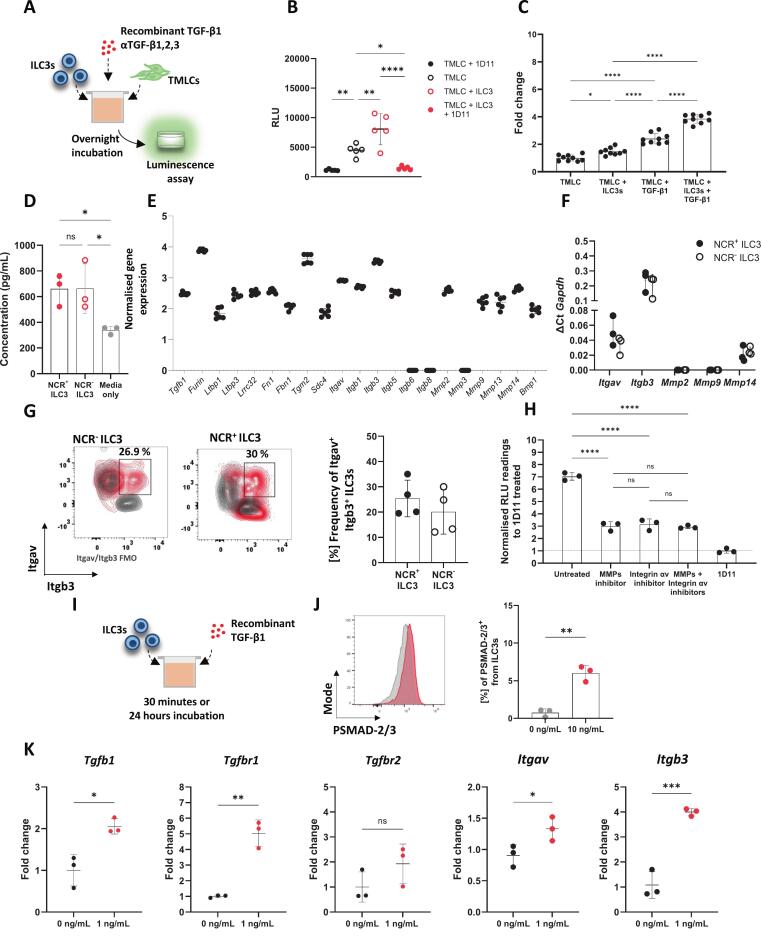
**Murine ILC3s activate TGF-β1 via integrins and metalloproteases.** (**A**) Schematic representation of experimental design: Lamina propria ILC3s (CD45^+^, Lin^-^, CD127^+^, KLRG1^-^, RORγt^+^, NK1.1^-^, NKp46^+/-^) were cultured with TMLC cells for approximately 17 h, followed by luminescence assay to detect active TGF-β1. (**B**) Relative light units (RLU) indicating active TGF-β1 levels in TMLC alone or co-cultured with ILC3s, with or without TGF-β1 neutralising antibody (1D11), *n* = 5. (**C**) Increase in active TGF-β1 post-stimulation with recombinant cytokine, *n* = 8. (**D**) Concentration of latent TGF-β1 detected from lamina propria extracted NCR^+^ and NCR^-^ ILC3s after stimulation with PMA/Ionomycin. (**E**) Dot plot showing normalised read counts of TGF-β1 activators in siLP ILC3s isolated from RORγt-GFP mice from a publicly available dataset^29^, *n* = 6. (**F**) *Itgav*, *Itgb3*, *Mmp2*, *Mmp9* and *Mmp14* expression in freshly isolated siLP NCR^+^ and NCR^-^ ILC3s detected via RT-qPCR, *n* = 3. (**G**) Flow cytometry of surface expression of Itgav and Itgb3 in NCR^+^ and NCR^-^ ILC3s. (**H**) RLU indicating active TGF-β1 levels after blocking TGF-β1 activators in co-cultures of TMLCs and ILC3s; data is normalised to the negative control, the horizontal dotted line (TMLC and ILC3 co-cultures treated with a TGF-β1 neutralising antibody), *n* = 3. (**I**) Schematic of ILC3s stimulated with recombinant TGF-β1 for 30 minutes or 24 hours. (**J**) Flow cytometry of SMAD-2/3 phosphorylation in 30 minutes stimulated (red) vs. unstimulated (grey) ILC3s, *n* = 3. (**K**) *Tgfb1*, *Tgfbr1*, *Tgfbr2*, *Itgav*, and *Itgb3* in untreated or 24 hours TGF-β1-treated ILC3s, *n* = 3. Data is represented as mean ± SD. Statistics: (**B**, **C**, **D**, **H**) one-way ANOVA, followed by Tukey’s multiple comparison test, (**J, K**) unpaired, two-tailed *t*-test. (ns = p > 0.05, *p < 0.05, ** p ≤ 0.01, *** p ≤ 0.001, **** p ≤ 0.0001). (For interpretation of the references to colour in this figure legend, the reader is referred to the web version of this article.)

To study if ILC3s produce and activate TGF-β1, small intestinal lamina propria (siLP) ILC3s (Supplementary Fig. 1A) were co-cultured with Transformed Mink Lung Cells (TMLC), which enable the detection of bioactive TGF-β1 as luciferase activity (Fig. 1A)^25^. This led to a significant increase in the levels of bioactive TGF-β1 in comparison to TMLC alone, an effect specifically blocked in the presence of a TGF-β1 neutralising antibody (1D11) (Fig. 1B). Addition of recombinant TGF-β1 augmented the levels of bioactive TGF-β1 in the co-cultures, potentially indicating enhanced ability of ILC3s to activate this cytokine (Fig. 1C). In contrast, whilst stimulation of ILC3s with activating cytokines IL-12/IL-18 and IL-23/IL-1β upregulated their expression of *Il22, Il17a* and *Ifng*, it did not augment activation of latent TGF-β1 (Supplementary Fig. 1B,C). Lastly, we tested the ability of ILC3s to produce latent TGF-β1 themselves and confirmed that in supernatant of PMA/Ionomycin stimulated NCR^+^ and NCR^-^ ILC3s the latent form of the cytokine was detected (Fig. 1D).

Immune and epithelial cells can activate TGF-β1 via αv-partnered integrins^16^, ^21^, ^22^, ^23^, ^24^, ^26^, ^27^, ^28^ and metalloproteases (e.g., MMP14^17^). Analysis of a publicly available bulk RNA sequencing dataset^29^ revealed that siLP ILC3s isolated from RORγt-GFP mice express the machinery required for TGF-β1 production and secretion, including *Tgfb1, Furin, Ltbp1* and *Ltbp3*^30^, TGF-β1 anchorage to the extracellular matrix (ECM) (*Tgm2, Sdc4*) as well as activation (*Itgav*, *Itgb1, Itgb3*, *Itgb5, Mmp9, Mmp14, Tgm2, Sdc4*, *Lrrc32*)^6^, ^19^, ^31^, ^32^ (Fig. 1E). Since *Itgb3*, was highly expressed and previously described to be expressed by LTi-like ILC3s^22^, we validated its expression together with *Itgav* and *Mmp14* in siLP ILC3s via RT-qPCR (Fig. 1F). Additionally, we observed co-expression of Itgav (CD51) and Itgb3 (CD61) on the cell surface (Fig. 1G), indicating that siLP ILC3 can express functional integrin αvβ3. Previous studies have indicated that emerging populations of non-ILC3 RORγt antigen presenting cells in the mesenteric lymph nodes (MLN) express *Itgb8* and perform TGF-β1-associated functions^23^, ^24^. However, our in silico analysis revealed that siILC3s lack the expression of *Itgb8* (Fig. 1E). To confirm this, we used a dual FoxP3-eGFP/Itgb8-tdTomato reporter mouse system^33^. We confirmed that siLP and cLP Tregs express Itgb8 as previously reported^21^, and verified that ILCs do not express Itgb8-tdTomato (Supplementary Fig. 1D). The TGF-β1-LAP complex may additionally be activated via the transmembrane protein Glycoprotein A Repetitions Predominant (GARP, encoded via *Lrr32*), as shown in Tregs where LAP can be detected on their extracellular membrane bound to GARP^34^. Although ILC3s express *Lrrc32,* LAP was only detectable on the surface of Tregs and not ILC3 (Supplementary Fig. 1E), suggesting that this pathway is not functional in ILC3s.

Next, we probed the capacity of siLP ILC3s to activate TGF-β1 via αv integrins and/or MMP14. Culture of siLP ILC3s with TMLC in the presence of either a broad-spectrum metalloprotease inhibitor (Ilomastat) or an αv inhibitor (Cilengitide), led to a decrease in the levels of active TGF-β1 (Fig. 1H), and did not impact cell viability (Supplementary Fig. 1F,G). The same decrease was observed when the inhibitors were used together, indicating that coordinated action between metalloproteases and integrin αv is necessary for optimal activation of latent TGF-β1.

Next, we examined the downstream effects of exogenous TGF-β1 stimulation in ILC3s (Fig. 1I). After 30 minutes, activation of the TGF-β1 canonical signalling pathway was determined via the increased levels of phosphorylated SMAD-2/3 (Fig. 1J). In addition, TGF-β1 stimulation for 24 hours led to the upregulation of genes downstream of TGF-β1 signalling (*Tgfb1*, *Tgfbr1*) as well as those involved in TGF-β1 activation (*Itgav*, *Itgb3*) (Fig. 1K) without affecting the viability of ILC3s (Supplementary Fig. 1H,I). Together, these data demonstrate that murine siLP ILC3s can produce TGF-β1 and possess conventional cellular machinery through which they can activate the latent form of the cytokine.

### ILC3-activated TGF-β1 induces FoxP3 expression in naïve T cells

We next determined the biological impact of ILC3-activated TGF-β1. TGF-β1 plays a critical role in driving differentiation and function of FoxP3^+^ Tregs. Furthermore, heterogeneous populations of antigen-presenting RORγt^+^ cells^35^ (including ILC3) have been shown to drive the expansion of intestinal microbiota-specific RORγt^+^ FoxP3^+^ Tregs in a manner dependent on MHC-II and αv integrins^22^, ^23^, ^24^. Thus, we decided to explore if ILC3s have the capacity to promote the induction of Treg differentiation via activation of TGF-β1^22^, ^23^, ^24^.

**Fig. 2:**
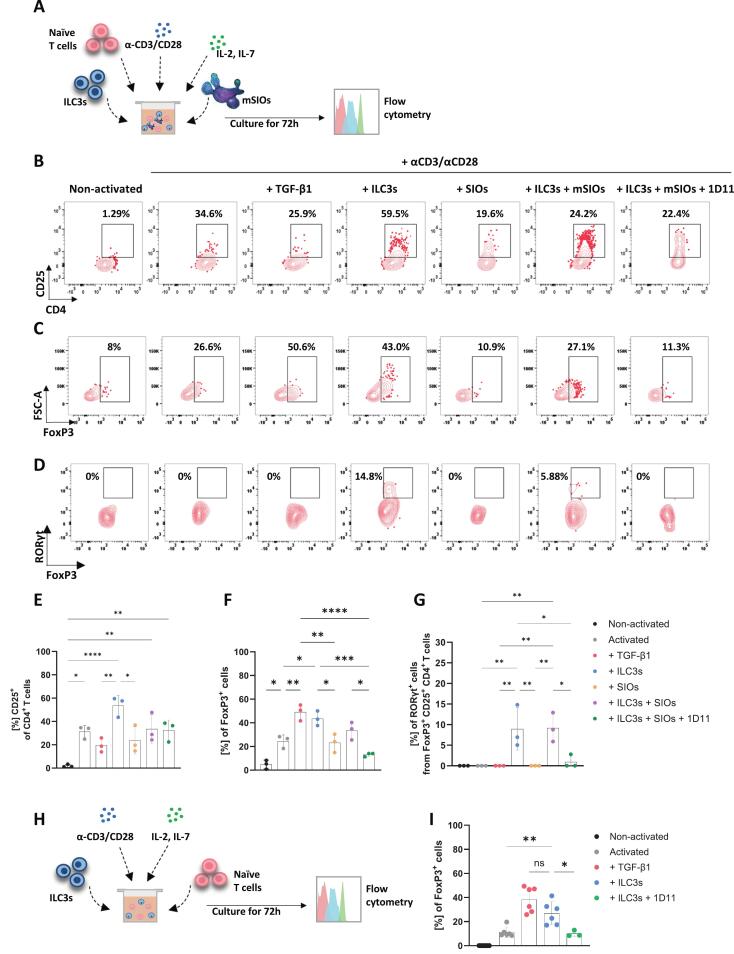
**siLP ILC3s activated TGF-β1 induce FoxP3 expression in naïve T cells.** (**A**) Schematic representation of experimental design in which ILC3s (CD45^+^, Lin^-^, CD127^+^, KLRG1^-^, RORγt^+^, NK1.1^-^, NKp46^+/-^) were co-cultured with murine small intestinal organoids (mSIOs) and naïve T cells (CD19^-^, CD25^-^, CD4^+^, CD44^low^, CD62L^+^) in the presence of IL-2, IL-7 and anti-CD3/CD28 antibodies for 72 hours with or without TGF-β1 neutralising antibody (1D11). (**B**) Representative flow cytometric plots of CD25^+^CD4^+^ T cells (pre-gated on live, EpCAM^-^, CD45^+^, CD3^+^, CD4^+^, CD44^+^) resulting from cultures described in (**A**), FoxP3^+^ from CD25^+^CD4^+^ T cells (**C**) and RORyt^+^ from FoxP3^+^ CD25^+^CD4^+^ T cells (**D**) populations within the CD25^+^ T cells shown in (**B**). (**E**) Frequency of activated T cells, followed by (**F**) the frequency of FoxP3^+^ T cells and (**G**) the frequency of RORγt^+^FoxP3^+^ from cultures described in (**A**), *n* = 3*.* (**H**) Schematic representation of experimental design in which ILC3s were co-cultured with naïve T cells in the presence of IL-2, IL-7 and anti-CD3/CD28 antibodies in Matrigel for 72 hours with or without TGF-β1 neutralising antibody (1D11). (**I**) Frequency of FoxP3^+^ T cells, n = 3–6. Data are represented as mean ± SD. Statistics: (**E**, **F**, **G, I**) one-way ANOVA, followed by Tukey’s multiple comparison test (* p ≤ 0.05, ** p ≤ 0.01, *** p ≤ 0.001, **** p ≤ 0.0001).

To better recapitulate what happens in vivo and recreate the intestinal microenvironment we established a novel tri-culture model where we cultured together murine intestinal organoids (mSIOs), naïve CD4^+^ T cells and siLP ILC3s (Fig. 2A and Supplementary Fig. 2A). T cell activation was achieved through CD3/CD28 cross-linking antibodies embedded within Matrigel. This produced the expected increased cell scatter (Supplementary Fig. 2B) and the upregulation of activation markers such as CD25 compared to T cells cultured without activating antibodies (Fig. 2, Fig. 2). Supplementation of the culture media with exogenous recombinant TGF-β1 promoted the differentiation of FoxP3^+^ T cells, confirming that induced Tregs (iTreg) could be derived from naïve T cells in a TGF-β1-dependent manner under these culture conditions (Fig. 2, Fig. 2). Remarkably, co-culture of naïve T cells with ILC3s induced FoxP3 expression and increased their expression of CD25. This indicates an increased state of T cell activation as a result of exposure to, or interaction with, ILC3s during the T cell activation process (Fig. 2, Fig. 2, Fig. 2, Fig. 2). FoxP3 expression was not observed when naïve T cells were activated in the presence of mSIOs only (Fig. 2, Fig. 2). Furthermore, neutralisation of TGF-β1 in the tri-culture model (Fig. 2, Fig. 2) as well as in a subsequent co-culture experiment involving ILC3s and naïve T cells alone (Fig. 2H,I) confirmed that the upregulation of FoxP3 expression in T cells was specifically driven by ILC3-derived TGF-β1. Together, these results confirm that within this system ILC3s are required for generation of FoxP3^+^ T cells, mediated via the production and/or activation of TGF-β1. We did not find this process to be influenced by either cell viability (Supplementary Fig. 2C, D) or through antigen presentation by ILC3s (Supplementary Fig. 2E, F).

The activation of naïve T cells in the presence of ILC3s additionally promoted the differentiation of RORγt^+^ FoxP3^+^ T cells (Fig. 2, Fig. 2). Critically, we demonstrated that this process was dependent on ILC3-derived TGF-β1, as inhibition of TGF-β signalling ablated these effects (Fig. 2, Fig. 2). However, the addition of exogenous TGF-β1 alone to naïve T cell cultures activated with CD3/CD28 cross-linking antibodies did not drive RORγt expression in FoxP3^+^ T cells (Fig. 2, Fig. 2), indicating that additional ILC3-derived factors are required.

Overall, these data show that in the tri-culture system and via exogenous T cell activation, siLP ILC3s can induce FoxP3 expression in T cells through their ability to activate TGF-β1, independently of ILC3 mediated MHC peptide-TCR interactions.

### ILC3-TGF-β1 axis induces TGF-β1 signalling in intestinal epithelial cells

Given that IECs can respond to active TGF-β1, competition for the active cytokine may underlie our observed dampening of ILC3-mediated induction of FoxP3^+^ T cells in the presence of mSIOs. Thus, we next determined the influence of active TGF-β1 on mSIOs.

**Fig. 3:**
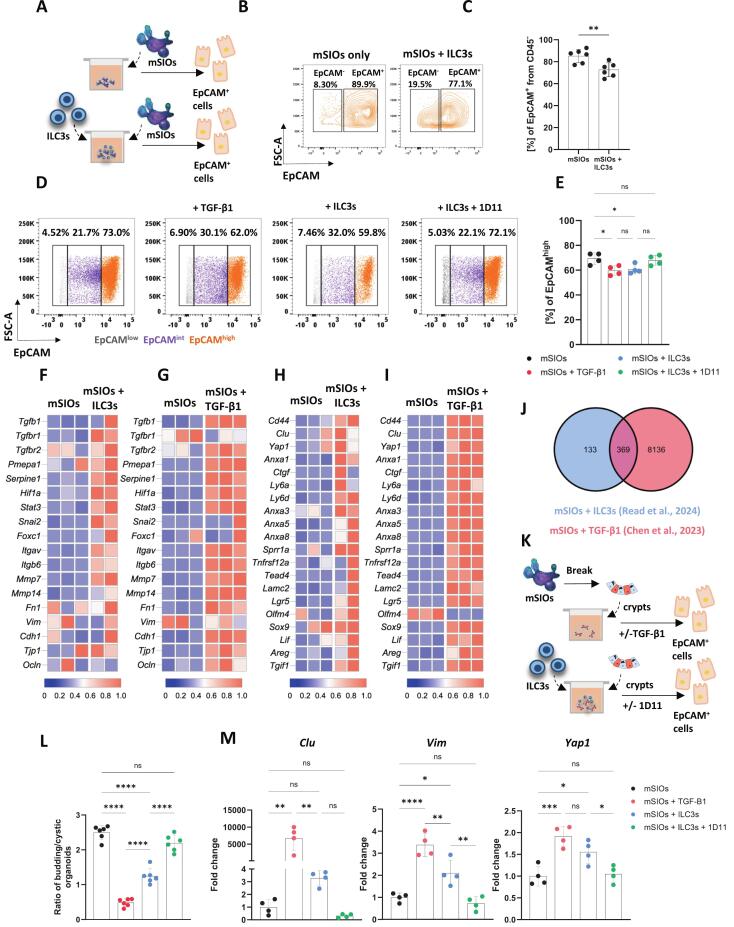
**ILC3 derived TGF-β1 induces TGF-β1 signalling in intestinal epithelial cells.** (**A**) Experimental design: ILC3s (CD45^+^, Lin^-^, CD127^+^, KLRG1^-^, RORγt^+^, NK1.1^-^, NKp46^+/-^) were co-cultured with murine small intestinal organoids (mSIOs) for 3 days followed by FACS sorting and analysis of epithelial cells (IECs). (**B**) Flow cytometric plots showing EpCAM expression in mSIOs cultured alone or with ILC3s. (**C**) Quantification of the extracellular levels of EpCAM on the surface of IECs, *n* = 6*.* (**D**) Flow cytometric plots of the distribution of EpCAM on IECs, and (**E**) quantification of EpCAM^high^ IECs, isolated from mSIOs cultured on their own, stimulated with TGF-β1, co-cultured with ILC3s, or co-cultured with ILC3s and a TGF-β1 neutralising antibody (1D11), *n* = 4. (**F-G**) Heatmaps of normalised re-analysed RNA-seq data showing genes related to EMT induction in IECs after mSIOs co-culture with ILC3s (**F**)^40^ and in IECs after mSIOs treatment with TGF-β1 (**G**)^10^. (**H-I**) Heatmaps of normalised re-analysed RNA-seq data showing genes related to regeneration in IECs after mSIOs co-culture with ILC3s (**H**)^19^, ^40^ and in IECs after mSIOs treatment with TGF- β1 (**I**)^10^. (**J**) Venn diagram showing the overlap of DEGs. The blue circle shows the number of DEGs from the mSIO + ILC3s co-culture dataset^40^ while the red circle displays the number of DEGs in the mSIO + TGF-β1 culture dataset^10^. (**K**) Experimental design: mSIOs were broken prior to experiment set-up. Crypts were cultured on their own, stimulated with TGF-β1, co-cultured with siLP ILC3s or co-cultured with siLP ILC3s and a TGF-β1 neutralising antibody (1D11). (**L**) The ratio of budding versus cystic mSIOs from cultures described in (Fig. 3K). Budding and cystic organoids were counted from 3 different planes of view per well**.***n = 6*. (**M**) *Clu, Vim* and *Yap1* expression in IECs isolated from mSIOs cultured under conditions described in (Fig. 3K), *n = 4*. Data are represented as mean ± SD. Statistics: (**C**) unpaired, two-tailed *t*-test, (**E, L, M**) one-way ANOVA, followed by Tukey’s multiple comparison test. (ns, non-significant = p > 0.05, * p ≤ 0.05, ** p ≤ 0.01, *** p ≤ 0.001, **** p ≤ 0.0001). (For interpretation of the references to colour in this figure legend, the reader is referred to the web version of this article.)

Co-culture of siLP ILC3s with mSIOs (Fig. 3A) led to downregulation of the epithelial cell marker Epithelial Cell Adhesion Molecule (EpCAM) on epithelial cells (CD45^-^ cells) (Figs. 3B, C), whilst the viability of the cultures remained unaffected (Supplementary Fig. 3A). EpCAM is required for the maintenance of intestinal epithelial architecture^36^ and its downregulation is a hallmark of epithelial to mesenchymal transition (EMT)^37^, a cellular process occurring under homeostatic conditions, including embryonic development, tissue regeneration, as well as in inflammation and pathological states such as cancer^38^. Interestingly, upstream pathways regulating EMT are associated with TGF-β1 signalling^39^. In line with this, addition of TGF-β1 neutralising antibody 1D11 to mSIO-ILC3 co-cultures prevented the downregulation of EpCAM, whilst treatment of mSIO monocultures with exogenous TGF-β1 recapitulated the EpCAM downregulation observed in the co-cultures with ILC3s (Figs. 3D, E). This suggested that the decrease in EpCAM was mediated via TGF-β1 in the presence of ILC3s. Furthermore, when mSIOs were either co-cultured with ILC3s or treated with exogenous TGF-β1, an EpCAM intermediate population was identified (Supplementary Fig. 3B), indicating the transitioning of epithelial cells towards an EpCAM^low^ phenotype (Figs. 3D, E). Using RT-qPCR, we confirmed that EpCAM^+^ cells isolated from both mSIO–ILC3 co-cultures, and mSIOs treated with exogenous TGF-β1, upregulated *Tgfb1, Tgfbr1, Tgfbr2, Itgav and Mmp14*, which was abrogated by TGF-β1 signalling inhibition (Supplementary Fig. 3C). Moreover, TGF-β1 stimulation augmented expression of these genes in line with the duration of the treatment (Supplementary Fig. 3D).

To further examine TGF-β1-induced transcriptomic alterations in mSIO epithelial cells following their co-culture with ILC3s, we analysed our bulk RNA sequencing dataset of mSIO cultures alone and co-cultured with ILC3^19^, ^40^. This showed that mSIOs upregulated genes and biological pathways associated with TGF-β1 induced signalling^41^ in the presence of ILC3s. Moreover, the epithelial cells displayed enhanced expression of genes downstream of TGF-β1 signalling, genes associated with EMT induction and EMT-associated transcription factors (Fig. 3F). Similar changes to the expression of EMT-associated genes were observed in a bulk-RNA sequencing dataset of organoids treated with recombinant TGF-β1^10^ (Fig. 3G). Considering EMT plays a key role in intestinal epithelial regeneration^42^ and that ILC3s, acting via YAP1 signalling pathway independent of IL-22, have been reported to drive regeneration of intestinal epithelial cells^43^, we explored this pathway. We observed that in the presence of siLP ILC3s, epithelial cells upregulated genes associated with regeneration, which had previously been shown to be induced by TGF-β1 signalling^10^ (Figs. 3H, I). Additionally, we found that the majority of differentially expressed genes (DEGs) previously identified in the mSIOs + ILC3s condition from the Read et al., 2024 dataset^40^ (369 out of 502) overlapped with those identified in the mSIOs + TGF-β1 condition from the Chen et al., 2023 dataset^10^ (Fig. 3J and Supplementary Table 1).

Given that siLP ILC3s upregulated genes associated with EMT and regeneration in IECs, we next investigated the impact of siLP ILC3s-derived TGF-β1 on organoid regeneration using a damage model^44^. Organoids were mechanically disrupted and then cultured with siLP ILC3s (Fig. 3K). After 48 hours, we quantified the number of budding versus cystic organoids and calculated their ratio (Fig. 3L). In both the siLP ILC3s co-culture and the TGF-β1 treatment condition, organoids predominantly exhibited a cystic morphology similar to what was previously described^10^. In contrast, when TGF-β1 was neutralised, the organoids retained their budding structure, similar to those cultured on their own (Fig. 3L). After the 48 hours of culture, the IECs were isolated from the cultures and we performed RT-qPCR (Fig. 3M). IECs from mSIOs co-cultured with siLP ILC3s or treated with exogenous TGF-β1 displayed increased expression of *Clu*, *Vim*, and *Yap1* genes previously associated with induction of EMT-like changes, intestinal regeneration and foetal reprogramming^10^, ^45^(Fig. 3M).

Lastly, to assess the in vivo relevance of these TGF-β1-dependent effects, we analysed a bulk RNA sequencing dataset^46^ (Supplementary Fig. 4) of epithelial cells from wild-type (WT) mice, mice lacking both Th17 cells and ILC3s (STOP), and mice lacking only ILC3s (ΔILC3). In both STOP and ΔILC3 mice, the expression of *Tgfb1* and *Mmp14* was downregulated compared to WT mice (Supplementary Fig. 4A) alongside TGF-β1-dependent genes involved in EMT pathways (i.e. *Foxc1*, *Hif1a*, *Stat3*, *Mmp7*) (Supplementary Fig. 4B) and epithelial regeneration (i.e. *Cd44*, *Clu*, *Ly6d*, *Anxa5*) (Supplementary Fig. 4C), supportive of our observations that ILC3s promote TGF-β1-driven transcriptional programs in IECs*.*

Together, these data identify a TGF-β1-mediated pathway of ILC3–IECs interactions which drives changes associated with a regenerative transcriptional signature in IECs.

### Human intestinal ILC3s express the machinery to activate latent TGF-β1 in health and disease

**Fig. 4:**
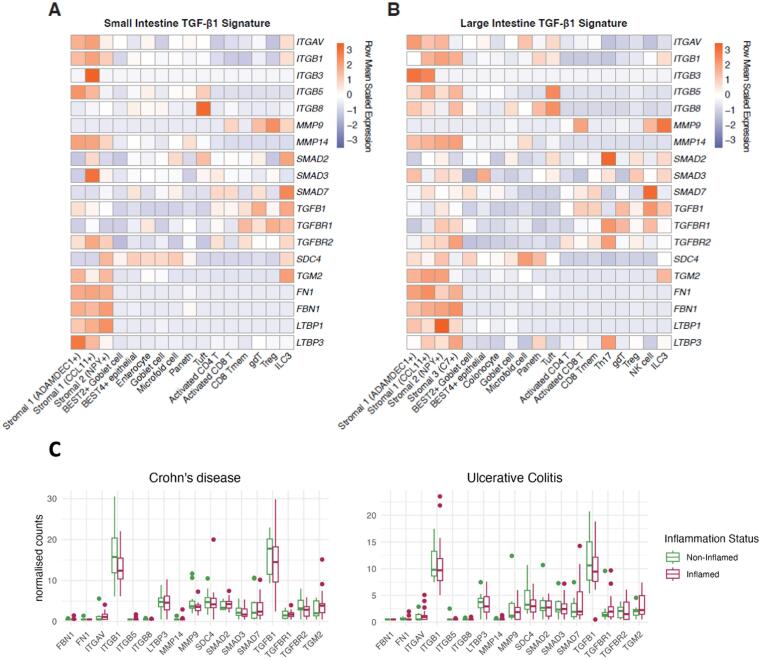
**Human intestinal ILC3s express the machinery to activate latent TGF-β1 in health and disease.** Relative expression of TGF-β1 signature genes from (**A**) small intestinal cells and (**B**) large intestinal cells, showing ILC3s expressing TGF-β1 activating machinery in conjunction with gut stromal, epithelial and lymphocyte populations (data re-analysed from The Gut Cell Atlas^47^. Average expression for each cell type is mean scaled by row to compare cell types per gene basis. (**C**) Expression of TGF-β1 signature genes in ILC3 isolated from inflamed and non-inflamed regions of the colon of individuals with CD or UC (re-analysed datasety. Data shown represents normalised counts of pseudobulked scRNA-seq data.

Next, we examined whether the capacity of ILC3s to activate TGF-β1 was conserved in humans. We assessed the expression of TGF-β1 activators across different cell types present in the small and large intestine using the Human Gut Cell Atlas^47^ (Fig. 4A). Mesenchymal, epithelial and adaptive T cells were included as comparators to ILC3s. Stromal cells exhibited the highest transcript levels for fibronectin (*FN1*) and fibrillin (*FBN1*), key proteins involved in ECM assembly and maintenance, as well as *LTBP1* and *LTBP3*, which are critical for the correct folding of TGF-β1 in the endoplasmic reticulum and its subsequent binding to the ECM^48^. While other cell types, including ILC3s, also expressed these genes, their levels were notably lower when compared to mesenchymal cells (Fig. 4A). Although ILC3s did not show high expression of genes associated with ECM deposition, *TGFB1* and its receptors (*TGFBR1* and *TGFBR2*) were clearly expressed, suggesting their ability to both produce and respond to TGF-β1. In addition, transcripts for *SMAD2*, *SMAD3* and *SMAD7* were detected in ILC3s, indicating their potential to engage in the canonical TGF-β1 signalling pathway. Additionally, both human (Fig. 4A) and murine ILC3s (Fig. 1E) expressed genes related to anchoring of TGF-β1 to the ECM, including *SDC4* and *TGM2*. Importantly human ILC3 also expressed genes involved in TGF-β1 activation, such as *ITGAV, ITGB1* and *MMP9*. Unlike in the murine model (Figs. 1D-F), we could not detect ITGB3 or *MMP14* in human ILC3s (Fig. 4A), suggesting mouse and human ILC3s may activate TGF-β1 via similar but differing members of integrin and metalloproteinase families.

Given the limited number of small intestinal ILC3s sequenced, we analysed human colonic ILC3s to increase the sample size and enhance the power of our findings (Fig. 4B). This revealed similar gene expression patterns related to TGF-β1 production and activation in colonic ILC3s. Th17 cells, the adaptive counterparts of ILC3s, expressed genes involved in both the production of TGF-β1 and its downstream signaling (including *SMAD2*, *SMAD3*, *TGFBR1*, *TGFBR2*, and *LTBP3*). However, they showed minimal expression of genes required for anchoring TGF-β1 to the ECM (*SDC4*, *TGM2*), as well as those involved in its mechanical (*ITGAV*, *ITGB1*, *ITGB3*, *ITGB5*, *ITGB8)* and proteolytic (*MMP9*, *MMP14*) activation (Fig. 4B) suggestive of decreased capacity to activate TGF-β1.

Next, we investigated the expression of these TGF-β1-related genes in the ILC3s-enriched cell population (Supplementary Fig. 5A) isolated from inflamed and non-inflamed intestinal regions in patients with CD and UC from a published dataset^49^ (Fig. 4C). No differences were observed in ILC3s isolated from either region (Fig. 4C), suggesting that the capacity of ILC3s to regulate TGF-β1 production and activation is not influenced by the inflammatory status of their microenvironment. To ensure the validity of these findings, we assessed the ability of ILC3s isolated from paediatric patients with CD from a published dataset^50^ to express these TGF-β1-related genes (Supplementary Fig. 5B). No differences in the expression levels of TGF-β1-related genes were observed in the ILC3s isolated from paediatric patients with CD (Supplementary Fig. 5B). To probe single cell transcriptomics findings, we analysed a bulk RNA sequencing dataset^51^ of ILC3s isolated from inflamed and non-inflamed regions of the colon in adult individuals with CD (Supplementary Fig. 5C) and UC (Supplementary Fig. 5D), which revealed similar results.

Lastly, we investigated whether adalimumab, an anti-TNF-α treatment, impacted the expression of these TGF-β1-related genes in the ILC3s-enriched cell population from inflamed and non-inflamed intestinal regions in IBD patients^49^ (Supplementary Fig. 6A). No differences were observed in ILC3s before or after adalimumab treatment in either CD or UC, suggesting that adalimumab does not affect the regulation of TGF-β1 machinery in ILC3s (Supplementary Fig. 6A). Together, these data suggest that the ability of ILC3s to produce and/or activate TGF-β1 in IBD is not altered during inflammation or in response to frontline monoclonal antibody therapy.

### Human intestinal ILC3s promote FoxP3 expression in naïve T cells via TGF-β1

As human ILC3s express *TGFB1* alongside transcripts for TGF-β1 activators, we next analysed TGF-β1-dependent ILC3s – T cells interactions in a human model. We adapted our intestinal ILC–organoid differentiation protocol^52^, ^53^, enabling the generation of large numbers of mature human intestinal ILCs from the co-culture of circulatory ILC precursors (ILCP) with human tissue-derived small intestinal organoids (hSIOs). This technique successfully differentiated ILCPs into ILC1s, ILC2s and ILC3s and these hSIO-maturated RORγt^+^ ILC3 produced their signature cytokines, IL-17A and IL-22 (Supplementary Fig. 7A-K).

**Fig. 5:**
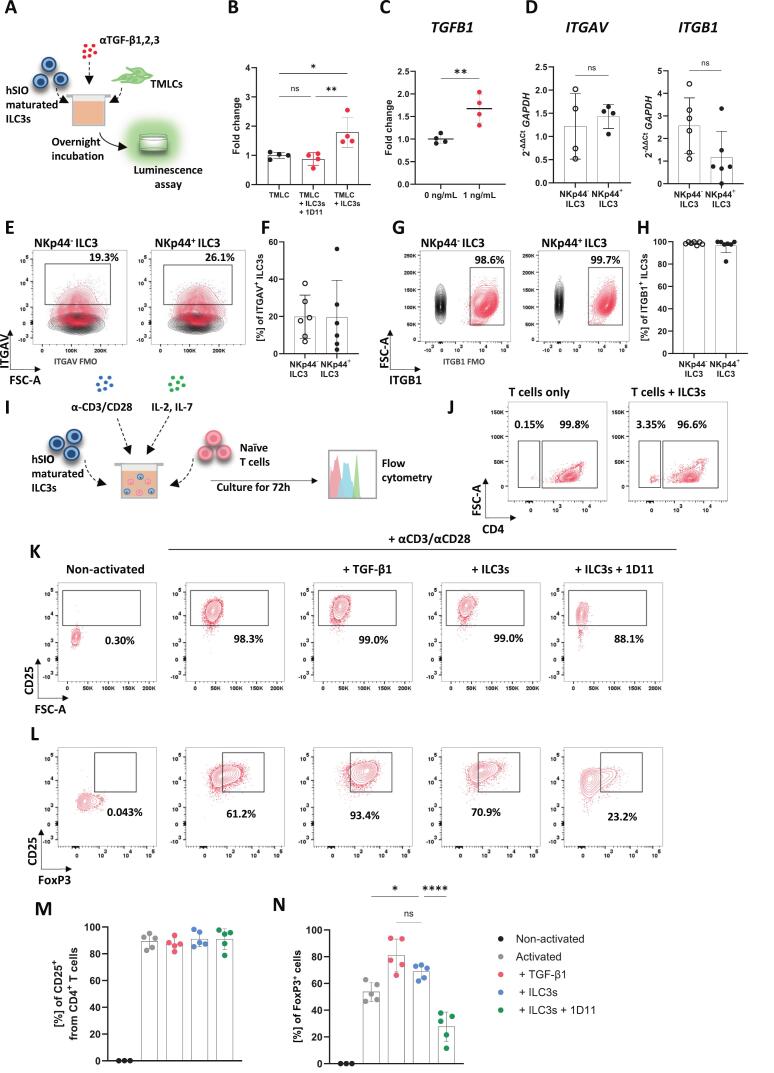
**Human small intestinal organoids-derived ILC3s induce FoxP3 expression in T cells via TGF-β1.** (**A**) Experimental design: human-SIO-matured ILC3s (EpCAM^-^, CD45^+^, Lin^-^, CRTh2^-^, cKIT^+^) were cultured with TMLCs for approximately 17 hours to detect active TGF-β1. (**B**) Relative light units (RLU) showing active TGF-β1 in TMLC cultures alone or with ILC3s, with or without the TGF-β1 neutralising antibody, 1D11, *n* = 4. (**C**) Fold change in *TGFB1* mRNA expression from ILC3s treated or untreated with recombinant TGF-β1, *n* = 4. (**D**) *ITGAV* and *ITGB1* relative gene expression from hSIOs-maturated ILC3s, *n* = 4. (**E**) Representative contour plots of ITGAV expression from NKp44^+/-^ ILC3s followed by (**F**) ITGAV quantification, *n* = 6. (**G**) Representative contour plots of ITGB1 expression from NKp44^+/-^ ILC3s followed by (**H**) ITGB1 quantification, *n* = 6. (**I**) Experimental design: hSIOs-maturated ILC3s were cultured with naïve T cells in the presence of cross-linking CD3/CD28 antibodies for 72 h. (**J**) Representative contour plots of activated T cells cultured on their own or co-cultured with hSIOs-maturated ILC3s. (**K**) Flow cytometric plots of CD25 expression in CD4^+^ T cells. (**L**) Flow cytometric plots of CD25^+^FoxP3^+^ expressing T cells. (**M**) CD25^+^CD4^+^ T cells quantification, *n* = 3–5. (**N**) CD25^+^FoxP3^+^ T cells quantification, *n* = 3–5. Data are represented as mean ± SD. Statistics: (**B, N**) one-way ANOVA, followed by Tukey’s multiple comparison test and Dunnett’s test respectively, (**C, D**) unpaired, two-tailed *t*-test. (ns, non-significant = p > 0.05, * p ≤ 0.05, ** p ≤ 0.01, **** p ≤ 0.0001).

Importantly, hSIO-matured ILC3s drove the activation of TGF-β1 (Fig. 5A, B) and treatment of hSIO-matured ILC3s with exogenous TGF-β1 resulted in the upregulation of *TGFB1* (Fig. 5C), indicating that TGF-β1 acts in an autocrine loop on hSIO-matured ILC3s to further promote the production of this cytokine. Moreover, hSIO-maturated ILC3s expressed both ITGAV and ITGB1 subunits of integrin αvβ1 at transcript (Fig. 5D) and protein levels (Fig. 5E-H). We also examined the expression of ITGB3, which was present in murine siLP ILC3s. However, hSIO-matured ILC3s did not express ITGB3 (Supplementary Fig. 7L). Similarly, hSIO-matured ILC3s, like their murine counterparts, did not express ITGB8 (Supplementary Fig. 7M, N).

To probe the ability of hSIO-matured ILC3s to induce FoxP3 expression in naïve T cells, we isolated naïve CD4^+^ T cells from peripheral blood and co-cultured them with autologous hSIO-matured ILC3s under anti-CD3/CD28 stimulation (Figs. 5I, J). T cell activation led to an increase in the proportion of CD25^+^ T cells compared to the non-activated control group (Figs. 5K, M). TCR activation via CD3/CD28 crosslinking alone was sufficient to induce a large degree of FoxP3 expression in human CD4^+^ T cells however addition of exogenous TGF-β1 significantly enhanced FoxP3 expression, as anticipated (Figs. 5L, N). Importantly, co-culture of hSIO-matured ILC3s with activated naïve T cells significantly increased FoxP3 expression relative to T cells activated in the absence of ILC3. The increase was mediated by the hSIOs-maturated ILC3-derived TGF-β1, as indicated by the significant reduction in FoxP3^+^ cells when TGF-β1 neutralising antibody was added (Figs. 5L, N). These findings support a role for ILC3s and activated TGF-β1 in promoting FoxP3 expression in human T cells.

### Human intestinal ILC3s drive transcript changes associated with TGF-β1 signalling in epithelial cells

**Fig. 6:**
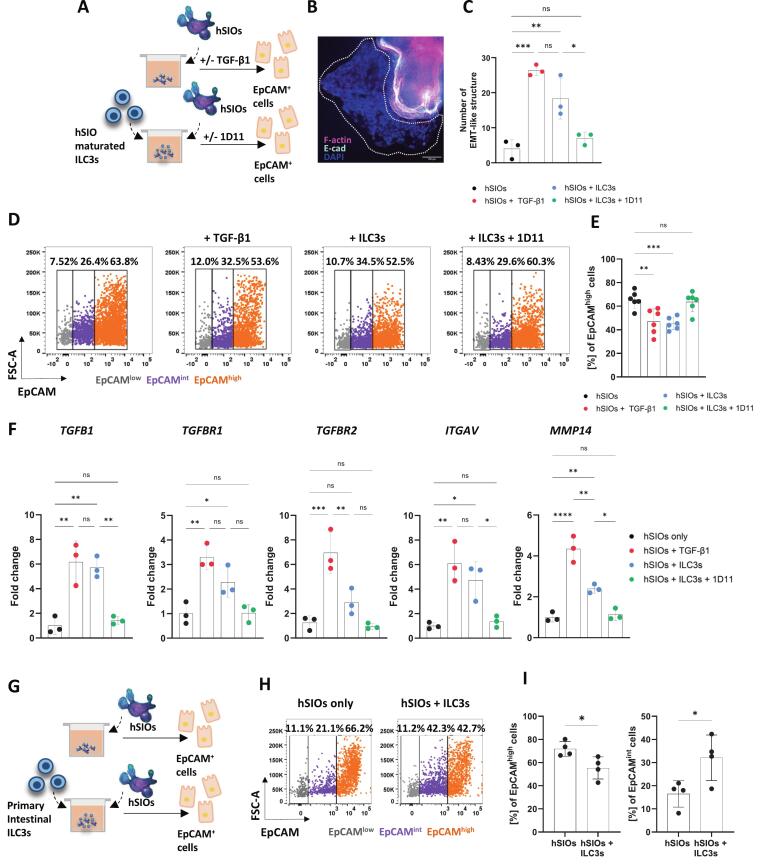
**TGF-β1 mediated interactions between human ILC3s and epithelial cells** (**A**) Experimental design: human-SIO were cultured on their own (with or without exogenous TGF-β1) and co-cultured with hSIOs-matured ILC3s (EpCAM^-^, CD45^+^, Lin^-^, CRTh2^-^, cKIT^+^) (with or without TGF-β1 neutralising antibody). (**B**) Visual representation of EMT-like structure (white dotted line) in maturated hSIOs co-cultured with ILC3s stained with Phalloidin – F-actin (magenta), DAPI – nucleic acid (blue) and E-cadherin (light-turquoise), scale bar = 100 μM. (**C**) Quantification EMT-like structures in organoids cultured under different conditions. *n = 3*. (**D**) Flow cytometric plots indicating the distribution of EpCAM on epithelial cells, followed by (**E**) quantification of EpCAM on the surface of epithelial cells isolated from organoids under various conditions, *n* = 6. (**F**) *TGFB1*, *TGFBR1*, *TGFBR2*, *ITGAV* and *MMP14* gene expression from epithelial cells isolated from hSIOs in single cultures, single cultures treated with recombinant TGF-β1, co-cultured with hSIOs-derived ILC3s, and co-cultured with hSIOs-derived ILC3s and TGF-β1inhibited (1D11), *n* = 3. (**G**) Experimental design: hSIOs were cultured on their own or with primary intestinal ILC3s (CD45^+^, Lin^-^ (CD3e, TCRα/β,TCRγ/δ, CD14, CD19, CD20), CD127^+^, CRTh2^-^, cKIT^+^). (**H**) Flow cytometric dot plots indicating low, intermediate and high EpCAM expression in epithelial cells from organoids cultured on their own or with primary intestinal ILC3s. (**I**) Quantification of high and intermediate EpCAM-expressing cells, *n* = 4. Data are represented as mean ± SD. Statistics: (**C**, **F**) one-way ANOVA, followed by Tukey’s multiple comparison test, (**E**) one-way ANOVA, followed Dunnett’s multiple comparison test, (**I**) unpaired, two-tailed *t*-test. (ns, non-significant = p > 0.05, * p ≤ 0.05, ** p ≤ 0.01, *** p ≤ 0.001, **** p ≤ 0.0001). (For interpretation of the references to colour in this figure legend, the reader is referred to the web version of this article.)

Next, we assessed the impact of activated TGF-β1 derived from hSIO-matured ILC3s on human IECs. To study this interaction, we purified ILC3s generated from our ILCP-hSIO co-cultures (Supplementary Fig. 7B) and re-seeded them with biopsy-derived hSIOs (Fig. 6A**,** and Supplementary Fig. 8A, B) either in the presence or absence of a TGF-β1 neutralising antibody. In parallel, hSIOs were treated with exogenous TGF-β1. Under both conditions—TGF-β1 treatment and co-culture with hSIO-matured ILC3s—the organoids exhibited morphological changes, forming EMT-like structures^54^ (Fig. 6B and Supplementary Fig. 8C). Quantification revealed a significant increase in the number of these structures when organoids were co-cultured with hSIO-matured ILC3s and exposed to TGF-β1. The number of these structures decreased upon addition of the neutralising antibody (Fig. 6C).

Further mirroring the findings in our mouse model, hSIO-matured ILC3s decreased the frequency of IECs expressing high levels of EpCAM (Figs. 6D, E) and increased the frequency of IECs with intermediate EpCAM expression (Supplementary Fig. 8D). We hypothesised that, similar to the mouse model, hSIO-matured ILC3s drive the observed changes in EpCAM expression through their production and/or activation of TGF-β1. Supporting this hypothesis, hSIOs cultured with exogenous TGF-β1 exhibited decreased EpCAM surface staining (Figs. 6D, E). Inhibiting active TGF-β1 signalling in the hSIO–ILC3 co-cultures prevented the loss of EpCAM on IECs (Figs. 6D, E). In IECs from hSIOs co-cultured with hSIO-matured ILC3, we also observed a TGF-β1-dependet upregulation of genes downstream of TGF-β1 signalling such as *TGFB1, TGFBR1, TGFBR2, ITGAV,* and *MMP14* (Fig. 6F). In addition, as observed in murine ILC3s-mSIOs cultures^40^, hSIO-matured ILC3s drove enrichment of epithelial secretory transcriptome signatures (*LYZ*, *MUC2*) and increased epithelial proliferation (*MKI67)* (Supplementary Fig. 8E). Moreover, hSIOs co-cultured with primary ILC3s isolated from healthy human small intestinal tissue biopsies also show decreased frequency of IECs expressing high levels of EpCAM, whilst correspondingly increased the frequency those with intermediate expression (Figs. 6G-I). These findings indicate that human ILC3s possess the capability for TGF-β1 production, activation and responsiveness and that ILC3-derived bioactive TGF-β1 influences the phenotype of intestinal epithelial cells, altering their transcriptional landscape and affecting downstream TGF-β1-dependent processes.

## Discussion

Innate and adaptive immune cells activate TGF-β1 through different mechanisms, including specific integrins^55^ and metalloproteases^17^, ^19^ Recent research shows that RORγt^+^ APCs, including ILC3s, express αv integrins, which have known roles in TGF-β1 activation.^22^, ^23^, ^24^ Here we show that ILC3s directly activate TGF-β1 and report the impact of this pathway in modulating T cell and epithelial function.

We demonstrate that both murine and human intestinal ILC3s express TGF-β1 on a transcriptomic level, in line with murine datasets.^56^, ^57^. Additionally, murine and human ILC3s express LTBPs (*Ltbp1, Ltbp3* / *LTBP3*), which are responsible for the assembly, secretion, and anchorage of latent TGF-β1 within the ECM, immobilising it onto fibrillin-rich microfibrils in the ECM via transglutaminase 2^58^ recruited by Syndecan 4^32^, ^59^. Human ILC3s were the only lymphocyte population examined in the small and large intestines with a high expression of both *SDC4* and *TGM2,* suggestive of a potential unique role of ILC3s in aiding TGF-β1 anchorage to the ECM. Murine ILC3s also express *Sdc4* and *Tgm2* transcripts indicating that ILC3-mediated mechanic activation of TGF-β1 occurs in both settings.

Using pan-inhibition of the integrin αv subunit and of MMPs, our results demonstrate that murine ILC3s activate TGF-β1 via these two components. Notably, we did not observe enhanced suppression of TGF-β1 activation when the two inhibitors were used in combination in comparison to their individual use. Additionally, we show that murine ILC3s express integrin αvβ3 and *Mmp14* transcripts, suggesting they mediate ILC3s activation of TGF-β1 and revealing a previously unreported coordinated pair of integrin/metalloproteases in ILC3s. Similar interactions are reported in macrophages where MMP14 and integrin αvβ8 are required for TGF-β1 activation^17^ while Tregs employ GARP alongside integrin αvβ8^60^ instead. The interaction between integrins and metalloproteases is therefore cell and context specific. That is also the case within the heterogenous RORγt-APCs population. Here we show that siLP ILC3s express integrin αvβ3 and not αvβ8 while previous reports have shown that Janus and Thetis cells express αvβ8^24^, ^25^. Whilst both mouse and human ILC3s activate TGF-β1, the components involved in the mechanism of activation differ between the species. Human small intestinal and colonic ILC3s lack the expression of all β-chain integrins, except for ITGB1 which was reported to drive TGF-β1 activation in tissue fibrosis^31^ while murine ILC3s express both *Itgb3* and *Itgb5* alongside *Itgb1.* In addition, human ILC3s express *MMP9* but not *MMP14.* Discrepancies between mouse and human TGF-β1 activation mechanisms have been reported in intestinal macrophages^17^ and highlight the importance of establishing human models to augment murine findings.

Active TGF-β1 plays multiple roles within the intestinal environment, influencing various immune cell types and is critical for Treg development. The capacity of RORγt^+^ APCs to support the in vivo and ex vivo expansion of Tregs and RORγt^+^ Tregs in mice has been documented^46^, ^51^, ^22^, ^23^, ^24^ but the mechanistic details still need to be elucidated. Investigating the specific impact of ILC3-derived TGF-β1 in vivo is challenging, given that TGF-β1 can be produced and activated by numerous cell types. Our tri-culture organoid model allows for the investigation of the ILC3 contribution. We demonstrate that murine and human ILC3s drive the differentiation of naïve T cells into FoxP3^+^ T cells through the activation of TGF-β1. This process is mediated in conjunction with IL-2 signalling, a cytokine secreted by ILC3s^61^, promoting the expression of the Treg master transcription factor FoxP3^62^. Thus, our data indicate that ILC3-derived bioactive TGF-β1 is capable of promoting FoxP3 expression in activated human T cells, highlighting the conservation of the potential immuno-regulatory roles for this ILC3-derived cytokine between the human and murine systems.

In addition, our findings reveal morphological, protein and gene expression changes in organoids cultured with ILC3s, suggesting that, through TGF-β1 production, ILC3 drive changes associated with regeneration, foetal reprogramming and EMT-like signalling in IECs. Key signalling pathways implicated in TGF-β1-induced EMT include Notch, YAP1/TAZ, and the TGF-β1 receptor complex^41^. Notch signalling is pivotal, as inhibition of Notch suppresses TGF-β1-induced EMT^63^. Our results show murine ILC3s upregulate Notch-associated genes (e.g., *Jag1* and *Psenen*) in IECs, highlighting the importance of this interaction^40^. Moreover, ILC3s can induce YAP1 in IECs, promoting intestinal epithelium repair independently of IL-22 signalling^43^. This suggests that another factor derived from ILC3s is involved. Given that YAP/TAZ signalling is upregulated in epithelial cells undergoing TGF-β1 mediated EMT^41^, this additional factor is likely to be TGF-β1. In line with this, we show that organoids undergoing TGF-β1 stimulation also upregulate YAP signalling. Together these findings indicate that ILC3s-derived TGF-β1 can mediate changes associated with EMT and intestinal regeneration.

Finally, our in vitro and in silico findings demonstrate that the ability of human ILC3s to activate TGF-β1 remains unaffected by the intestinal pro-inflammatory microenvironment in both CD and UC. Our findings indicate that novel strategies targeting the ILC3–TGF-β1 axis could play a crucial role in promoting intestinal homeostasis. Given that ILC3s are largely depleted in IBD^5^, we would anticipate that their TGF-β1-mediated protective functions would also be limited. Future therapeutic strategies could aim to prevent ILC3s depletion, to promote their expansion or to adoptive transfer intestinal organoid-generated autologous ILC3s to promote epithelial regeneration and restore intestinal homeostasis.

## Materials and methods

### Animals

All animal experiments were performed at the New Hunt’s House Biological Services Unit, King’s College London, in compliance with the UK Animals (Scientific Procedures) Act 1986 (UK Home Office licenses PPL 70/7869, PP7740871 and PP8070044). Male and female Rorc(γt)-GFP (C57BL/6J) reporter mice (6–12 weeks old), kindly provided by Gerald Eberl^64^, were used to isolate lamina propria ILC3s. FoxP3-eGFP/Itgb8-IRES-tdTomato reporter mice^33^ were used to assess Itgb8 expression in LP ILC3s.

### Murine organoid generation and maintenance

Murine SIOs were generated from the duodenum using a standard protocol^65^ (for further details, see online supplemental materials and methods). Once established, the organoids were passaged every 4–6 days by mechanical disruption.

### Isolation and ex vivo culture of mature ILC3s from mouse lamina propria

Lamina propria lymphocytes were isolated following established protocols^66^ (for detailed instructions, see online supplemental materials and methods).

### ILC3–mSIO co-culture set-up

ILC3 were isolated from the lamina propria and co-cultured with mSIO using our published protocol^65^ (for further details, see online supplemental materials and methods).

### ILC3–mSIO–Naïve T cell tri-culture set-up

Approximated 100 mSIOs were combined with ∼ 1 × 10^4^ ILC3s and ∼ 5 × 10^4^ naïve T cells per condition in Matrigel supplemented with 2 µg/mL of anti-CD3 and anti-CD28 for T cell activation. The cultures were fed with complete co-culture medium (Table 2). FoxP3+ T cell induction was achieved with 2 ng/mL of recombinant TGF-β1 (for further details, see online supplemental materials and methods).

### Human tissue samples

All human samples were acquired after informed consent and in compliance with local ethical approval (Research Ethics Committee numbers: 16/LO/0642 and 15/LO/1998) (for further details on patient recruitment, see online supplemental materials and methods). Patients and the public were not involved in the design, conduct, or reporting of this research and will be involved in dissemination plans.

### Lymphocyte isolation from small intestinal biopsies

Whole small intestinal biopsies were digested using human Tumour Dissociation Kit (Miltenyi Biotec) according to manufacturer’s instructions for the isolation of lymphocytes. Prior to sorting, the isolated cells were rested for approximately 17 hours in RPMI 1640 (supplemented with 10 % foetal calf serum, β-mercaptoethanol, penicillin (100U/ml), streptomycin (100 μg/mL), metronidazole (1 μg/mL), gentamicin (20 μg/mL), amphotericin (2.5 μg/mL), IL-2 (200U/mL) and IL-15 (20 ng/mL)). ILC3s were FACS isolated based on the following markers: CD45^+^, Lin^-^ (CD3e, TCRα/β,TCRγ/δ, CD14, CD19, CD20), CD127^+^, CRTh2^-^, cKIT^+^.

### Biopsy derived human intestinal organoid (hSIO) generation, maintenance and maturation

Human SIOs were generated following a published protocol^67^ (for further details, see online supplemental materials and methods). The cultures were fed with complete Intesticult Organoid Growth Medium (OGM) (Table 2) and split every 10–14 days. Organoids were differentiated with complete Organoid Differentiation Medium (ODM) (Table 2) supplemented with 5 mM DAPT, for further details, see online supplemental materials and methods.

### Innate lymphoid cell precursors maturation using hSIOs

FACS-isolated ILCPs were combined with hSIOs derived from a different donor in Matrigel. Co-cultured were fed with human co-culture medium (Table 2). ILCPs were expanded for 21–28 days at which point the hSIO-derived ILC3s were isolated via FACS, for further details see the online supplemental materials and methods.

### hSIOs-derived ILC3s-T cells co-culture set-up

Naïve T cells and hSIOs-maturated ILC3s derived from the same donor were FACS-isolated and combined in Matrigel – supplemented with 2 µg/mL of anti-CD3 and anti-CD28 for T cell activation. FoxP3^+^ T cell induction was achieved with 2 ng/mL of recombinant TGF-β1. Once Matrigel solidified, human co-culture medium was added (Table 2), for further details, see online supplemental materials and methods.

### TMLC cell culture maintenance and assay set-up

Transformed mink lung cells (TMLC) were cultured in TMLC medium (Table 2), incubated at 37 °C with 5 % CO_2_ and passaged at 80 % confluency. To assess ILC3 activation of TGF-β1, 1 × 104 ILC3s were co-cultured with 1.6 × 104 TMLCs in 200 µL of complete DMEM medium (Table 2). After 20 hours of incubation at 37 °C and 5 % CO_2_, a luciferase assay (Promega E1501) was performed and luminescence measured, for further details, see online supplemental materials and methods.

### Phenotyping by flow cytometry

Cells were stained with fixable LIVE/DEAD dye (ThermoFisher) and then washed. Fc receptors were blocked (anti-CD16/32) before the cells were incubated with extracellular antibody cocktails (all antibodies used are listed in Table 3). After washing, cells were resuspended in FACS buffer (Table 1) for immediate analysis. For intracellular staining, cells were washed in PBS2, fixed and permeabilised using the FoxP3 Intracellular Staining Kit (Invitrogen). Cells were stained for intracellular cytokines and transcription factors and samples were analysed using a BD LSRFortessa II Cell Analyzer. For SMAD-2/3 phosphorylation assay see online supplemental materials and methods.

### Gene expression quantification via RT-qPCR

Cells were lysed in 300 µL RLT buffer (with 10 µL/mL BME). RNA was extracted following the RNeasy micro kit (Qiagen) protocol and cDNA was synthesised from mRNA using the RevertAid First Strand cDNA kit (ThermoFisher). RT–qPCR based on Fast SYBR Green Mix (Applied Biosystems) or using TaqMan Gene Expression Master Mix (Applied Biosystems) was run ViiA 7 Real-Time PCR System (Applied Biosystems). The relative expression of each gene used was calculated using the comparative cycle threshold (Ct) method (2-ΔΔCt) using *Hprt1* or *GAPDH* for SYBR primers (Table 5) and *Gapdh* or *GAPDH* for TAQ probes (Table 6).

### Organoid whole mount immunostaining

Cells were fixed in PFA. Epitopes were unmasked by incubating organoids in staining buffer (Table 1). Samples were blocked with blocking solution (Table 1) before incubation with the primary antibodies (Table 7) for approximately 17 hours at 4 °C. Cultures were washed and incubated with the secondary antibodies (Table 7) for 1 hour at room temperature in the dark. Imaging was performed using a THUNDER Imager Live Cell & 3D Assay.

### Single cell-RNAseq data acquisition, availability and processing

The Space-Time Gut Cell Atlas dataset (ST-GCA), the paediatric IBD (pIBD) and adalimumab IBD data (adaIBD) detailed sample collection and data generation information is available from the original publications^47^, ^49^, ^50^. For detailed information on the quality control and processing of the ST-GCA, pIBD and adaIBD see online supplemental materials and methods.

### LAP quantification by ELISA

SiLP ILC3s were FACS isolated from RORγt-GFP mice and placed in culture overnight. The following day, they were stimulated for 4 hours with 1 μg/mL phorbol myristate acetate (PMA) (P1585, Sigma-Aldrich) and 100 ng/mL of Ionomycin (I0634, Sigma-Aldrich). The cells were then harvested, spun down and the supernatant used for LAP quantification. The LAP ELISA (Invitrogen, cat number: 88–50690-22) was performed following manufacturer’s recommendations.

### Analysis of bulk RNA-seq datasets

For the analysis of published bulk RNA sequencing datasets, the raw count matrices were downloaded from Gene Expression Omibus (GEO accession numbers: GSE243737 (Fig. 3F,H,J), GSE85154 (Fig. 1E), and GSE270398 (Supplementary Fig. 4)). The data for the mSIOs only controls analysed together with the GSE243737 dataset was deposited under the Sequence read archive numbers: SRX8615003, SRX8615004 and SRX8615005. For the GSE222505 (Fig. 3G,I,J) the raw sequencing reads were downloaded and pre-processed (for detailed information, see supplemental materials and methods). These counts were imported into GraphPad Prism (10.3.1 (686)) normalised via log10 transformation and heatmaps were generated following min–max scaling of the normalised read counts.

### Statistical analysis

Data was analysed using Microsoft Office 365 Excel (version 2407) and GraphPad Prism 10.3.1 (686). Flow cytometric analysis was performed using FlowJo 10.4.1 (for further details, see online supplemental materials and methods).

## Credit authorship contribution statement

**Diana Coman:** Writing – review & editing, Writing – original draft, Visualization, Validation, Project administration, Methodology, Investigation, Formal analysis, Conceptualization. **John W. Bassett:** Writing – review & editing, Visualization, Investigation. **Isabelle Coales:** Writing – review & editing, Visualization, Investigation. **Ainize Peña-Cearra:** Writing – review & editing, Investigation. **Emily Read:** Writing – review & editing, Investigation. **Emma H. Kromann:** Investigation. **Daniel Brice:** Investigation. **Zuzanna Łukasik:** Writing – review & editing, Investigation. **Helena Paidassi:** Investigation. **Matthew R. Hepworth:** Writing – review & editing, Methodology. **Robin J. Dart:** Writing – review & editing, Resources, Investigation. **Mark A. Travis:** Writing – review & editing, Resources, Methodology. **Jenny Mjösberg:** Writing – review & editing, Supervision, Funding acquisition. **Luke B. Roberts:** Writing – review & editing, Writing – original draft, Supervision, Methodology, Investigation, Conceptualization. **Joana F. Neves:** Writing – review & editing, Writing – original draft, Supervision, Project administration, Methodology, Investigation, Funding acquisition, Conceptualization.

## Funding

D.C., I.C. and J.F.N. acknowledge funding support by The Leona M. and Harry B. Helmsley Charitable Trust. In addition, D.C. acknowledges a PhD studentship from the National Institute of Health and Care Research (NIHR) BRC based at Guy’s and St. Thomas’ (GSTT) NHS Foundation Trust and King’s College London (KCL). J.F.N acknowledges funding from The Lister Institute of Preventive Medicine and a RCUK/UKRI Rutherford Fund fellowship (MR/R024812/1). E.R. acknowledges a Ph.D. fellowship from the Wellcome Trust (215027/Z/18/Z). A.P.C. acknowledges funding by the Basque Government. E.H.K. acknowledges Ph.D. funding from the Leverhulme Trust and KCL as part of the Mechanics of Life Leverhulme Doctoral Scholarship Programme. L.B.R. acknowledges funding by NIHR BRC based at GSTT and KCL. J.M. is funded through the Swedish Research Council, the Swedish Cancer Foundation, The Knut and Alice Wallenberg Foundation, Karolinska Institutet and the European Research Council (ERC) under the Horizon 2020 research and innovation programme (Grant agreement No. 850963). M.A.T acknowledges funding from the MRC (MR/V011243/1). M.R.H. is supported by a Wellcome Trust Career Development Award (CDA; 227760/Z/23/Z).

## Declaration of competing interest

The authors declare that they have no known competing financial interests or personal relationships that could have appeared to influence the work reported in this paper.
