# Supplementary material for: ILC3s mediate intestinal immune-epithelial interactions via TGF-β1 activation
Source: Mucosal Immunol. 2026 Apr;19(2):1735–48. doi: 10.1016/j.mucimm.2025.11.013 (PMC13195368; doi:10.1016/j.mucimm.2025.11.013)
Supplement: Supplementary Data 1 [file mmc1.pdf]

## SUPPLEMENTAL MATERIALS AND METHODS

### Animals

Animals were housed in specific-pathogen free conditions with controlled temperature (20-24°C), humidity (45-65% RH), and a 12/12-hr light/dark cycle. Female wild-type C57BL/6J mice (6-8 weeks old) were used to generate small intestinal organoids (mSIOs).

### Murine organoid generation and maintenance

In brief, the duodenum was cut longitudinally, cleaned and washed in phosphate-buffered saline (PBS) (Gibco). Next, 2-3 mm segments were incubated in crypt isolation buffer (**Table 1**) at 4°C for 30 minutes, whilst rocking, before transfer to PBS. Approximately 500 crypts were plated in Matrigel (Corning) onto a pre-warmed 24-well plate and then fed with EGF-Noggin-R-spondin (ENR) medium (**Table 2**) once the Matrigel solidified to form organoid cultures.

### Isolation and ex vivo culture of mature ILC3s from mouse lamina propria

Following the isolation of the small intestine from 6-8-week-old mice, the external fat and Peyer's Paches were removed before the tissue was cleaned and cut into 1 cm pieces. After washing in PBS, the tissues were incubated in epithelium removal buffer (**Table 1**) for 30 minutes at 37°C in the shaking water bath. They were then minced and placed in digestion buffer (**Table 1**) for a further 30 minutes at 37°C in the shaking water bath. The digested tissue was filtered and enzyme activity was stopped with DMEM (Gibco) + 10% FCS. Following centrifugation, immune cells were enriched using an 80%/40% isotonic Percoll (**Table 1**) (45-001-747, Fisher Scientific) gradient. The ILC3s were sorted using a BD FACS ARIA II (BD Biosciences) based on expression of the following markers: $CD45^+$, $CD3^-$, $CD19^-$, $CD5^-$, Gr-$1^-$

, TER-19$^-$, CD127$^+$, KLRG1$^-$, ROR$\gamma$t$^+$, NK1.1$^-$ and NKp46$^{+/-}$ (**Table 3**). FACS isolated ILC3s were cultured with complete DMEM medium (**Table 2**).When the ability of ILC3s to produce specific cytokines was assessed, ILC3s were stimulated for approximately 17 hours with 12.5 ng/mL phorbol myristate acetate (PMA) (P1585, Sigma-Aldrich) and 166.66 nM of Ionomycin (I0634, Sigma-Aldrich) or with IL-12/IL-18 and IL-1β/IL-23 (**Table 4**) for approximately 17 hours.

**ILC3–mSIO co-culture set-up**

Organoids were split 24 hours in advance of their removal from Matrigel, at which point they were washed in Advanced DMEM/F12 (Fisher Scientific) and gently resuspended. FACS-purified ILC3s were added to the organoids (at approximately 200 ILC3s per organoid). The mixture was then centrifuged (300g / 4°C / 5 mins), the supernatant removed, and the cells resuspended in Matrigel. Once solidified, complete co-culture medium (**Table 2**) was added.

**ILC3–mSIO–Naïve T cell tri-culture set-up**

Naïve T cells were sorted from C57BL/6J splenocytes. Spleens were harvested in ice-cold PBS, passed through a 40 μM strainer and washed with PBS. The cell suspension was centrifuged (300g / 4°C / 5 mins) and treated with ACK lysis buffer (Life Technologies Ltd.). Lysis was stopped with an excess of PBS. The cells were sorted using a BD FACS ARIA II based on the following extracellular markers (CD19$^-$, CD25$^-$, CD4$^+$, CD44$^{low}$, CD62L$^+$) (**Table 3**). Organoids were split 24 hours in advance of their removal from Matrigel, after which they were washed, and 100 organoids were combined with ~$1\times10^4$ ILC3s and ~$5\times10^4$ naïve T cells per condition. The mixture was centrifuged (300g / 4°C / 5 mins) and the cells resuspended in Matrigel – supplemented with 2 μg/mL of anti-CD3 (clone:145-2C11, 100302, BioLegend) and anti-CD28 (clone: 37.51, 102101, BioLegend) for T cell activation. Once solidified, the

cultures were fed with complete co-culture medium (**Table 2**). $FoxP3^{+}$ T cell induction was achieved with 2 ng/mL of recombinant TGF-β1 (240-B-002, Bio-Techne).

### Human tissue samples

Endoscopic biopsies were obtained from the macroscopically normal terminal ileum of non-IBD control adult donors undergoing routine diagnostic ileocolonoscopy for non-inflammatory indications including rectal bleeding or polyp surveillance. Patients with coeliac disease or other significant intestinal pathology were excluded.

### Biopsy derived human intestinal organoid (hSIO) generation, maintenance and maturation

Between 5-10 small intestinal biopsies were collected in PBS, cut into 1-2 mm pieces and washed with additional PBS. Next, the tissue segments were rotated at 15 RPMI, 4°C for 1 hr in chelation buffer (**Table 1**) before inactivation with PBS containing $Ca^{+}$ $Mg^{+}$. The detached crypts were then centrifuged (300g / 4°C / 5 mins) and resuspended in Matrigel; once solidified, complete Intesticult Organoid Growth Medium (OGM) (STEMCELL Technologies) was added. The cultures were passaged every 10 days via incubating at 37°C with TryplE for 3 minutes, followed by the addition of neutralisation buffer (**Table 1**). The cells were then spun down, resuspended in Matrigel, and fed with complete OGM (**Table 2**). For differentiation, organoids were passaged five days prior, replated in Matrigel, and fed with complete Organoid Differentiation Medium (ODM) (**Table 2**) (STEMCELL Technologies) supplemented with 5mM DAPT (2634/10, Tocris) for 5-7 days.

### Innate lymphoid cell precursors maturation using hSIOs

PBMCs (NHS-BT) were thawed and washed with 1X PBS before staining for ILCP purification via a BD FACS ARIA II based on the following markers CD45$^{+}$, Lineage (CD14, CD19, CD20, CD3, TCRαβ, TCRγδ)$^{-}$, CD127$^{+}$, KLRG1$^{-}$, CD4$^{-}$, NKp46$^{-}$, CD56$^{-}$, CD294$^{-}$, CD117$^{+}$) (**Table 3**). Four days following their last passage, organoids were removed from Matrigel, washed in Advanced DMEM/F12 and gently resuspended. Approximately 100 organoids were combined with 1,000 FACS-purified ILCPs per well before centrifugation (300g / 4°C / 5 mins) and resuspension in Matrigel. Once solidified, human co-culture medium (**Table 2**) was added. ILCPs were expanded for 21-28 days at which point the hSIO-derived ILC3s were isolated via FACS.

**hSIOs-derived ILC3s-T cells co-culture set-up**

PBMCs (NHS-BT) were thawed and washed with 1X PBS before staining for naïve T cell purification via a BD FACS ARIA II based on the following markers CD4$^{+}$,CD8$^{-}$, CD127$^{+}$, CD25$^{-}$, CD45RA$^{+}$, CD62L$^{+}$ (**Table 3**). hSIOs-maturated ILC3s were removed from co-cultures after 21-28 days of expansion and isolated via FACS based on the following markers: CD45$^{+}$, (CD14, CD19, CD20, CD3, TCRαβ, TCRγδ)$^{-}$, CD56$^{+/-}$, CD294$^{-}$, CD117$^{+}$, NKp44$^{+/-}$. Approximately $5\times10^4$ naïve T cells and $1\times10^4$ hSIOs-maturated ILC3s were combined and centrifuged (300g / 4°C / 5 mins). The cells were resuspended in Matrigel – supplemented with 2 µg/mL of anti-CD3 (clone: OKT3, 14-0037-82, eBioscience™) and anti-CD28 (clone: CD28.2, 16-0288-81, eBioscience™) for T cell activation. Once solidified, the cultures were fed with complete co-culture medium (**Table 2**). FoxP3$^{+}$ T cells induction was achieved with 2 ng/mL of recombinant TGF-β1 (240-B-002, Bio-Techne). Once Matrigel solidified, human co-culture medium was added (**Table 2**).

**End point cultures dissociation into single cells for downstream analysis**

Murine and human organoids single cultures, co-cultures and tri-cultures were processed similarly. Matrigel domes were dislodged using P-1000 tip and transferred to a PBS2 (**Table 2**)-coated 15 mL tube. Cells were spun down (300g / 4°C / 5 mins) and then resuspended in 250 µL pre-warmed TryplE (12604013, Thermofisher) and incubated for 10-15 min at 37°C. Dissociation was stopped with 1 mL PBS2, and the cells were filtered through a 100 µm strainer for downstream analyses.

**TMLC cell culture maintenance and assay set-up**

To passage TMLCs, 0.25% Trypsin-EDTA (11570626, Fisher Scientific) was added for two minutes before being neutralised with PBS10 (**Table 1**). The cells were centrifuged (400g / 4°C / 4 mins) and re-plated with fresh TMLC medium (**Table 2**) and supplemented with 10 µL/1 mL of G418 sulphate (Merck). For TMLC assay set-up, TMLCs were harvested as described above. $1.6\times10^4$ TMLCs were plated per well in a 96-well plate and incubated for 3 hours to allow the cells to attach and acclimatise. To assess ILC3 activation of TGF-β1, $1\times10^4$ ILC3s were co-cultured on top of the pre-existing $1.6\times10^4$ TMLCs in 200 µL of complete DMEM medium (**Table 2**). For negative controls, TGF-β1,2,3 neutralising antibody (1D11, 40 µg/mL) was added. After 20 hours of incubation at 37°C and 5% $CO_2$, a luciferase assay (Promega E1501) was performed. TMLCs were washed in PBS and lysed (5X Lysis Reagent, Promega). Luminescence was measured using the GloMAX®-Multi Detection System.

**SMAD-2/3 phosphorylation assay**

ILC3s were seeded in complete DMEM medium (**Table 2**) with 1% FBS and rested for approximately 17 hours. The following day, cells were stimulated with 10 ng/mL recombinant TGF-β1 (Bio-Techne). To minimise handling, 100 µL of Live/Dead dye was added and gently resuspended. For extracellular marker analysis, antibodies were added at this point and the cells

were incubated for 30 minutes at 37°C and 5% $CO_2$. The cells were then incubated in pre-warmed Lyse/Fix buffer (BD PhosflowTM, BD Biosciences) for 10 minutes at 37°C. Next, the cells were washed in PBS and incubated in Permeabilization Buffer III (BD PhosflowTM, BD Biosciences) on ice for 30 minutes. After washing with PBS, the cells were stained with P-SMAD-2/3 antibody in FACS buffer for 20 minutes at 4°C. Samples were washed and resuspended in FACS buffer for analysis.

**Quantification of budding vs cystic organoids ratio**

Murine organoids were seeded in triplicate wells for each experimental condition. For each well, three representative images were captured, from the left, centre, and right regions. Organoids were classified as either budding or cystic in each image, and the counts were averaged across the three views to obtain a representative number of budding and cystic organoids per well. The ratio of budding to cystic organoids was then calculated by dividing the total number of budding organoids by the total number of cystic organoids.

**Sample information**

**ST-GCA:** Samples from the Space-Time Gut Cell Atlas used in this analysis comprise tissue samples from six deceased adult organ donors and are split into the regions of the small and large intestine. The large intestine data used comprises 38 total 10X experiments across five regions including the caecum, ascending colon, transverse colon, descending Colon, and sigmoid colon. The small intestine data used comprises 19 total 10x experiments across three regions including the duodenum, jejunum, and ileum.

**pIBD:** Sample data used from the paediatric IBD dataset comprises 16 biopsies from 19 total patients of which 10 biopsies are characterised as non-inflamed controls and 6 are characterised as inflamed by endoscopy.

**Data acquisition and availability**

The Space-Time Gut Cell Atlas dataset (**ST-GCA**) is made up of 10x Genomics SC-RNAseq data using Chromium Single-Cell 5' or 3' gene expression v2 reagents. The raw and normalised single-cell RNA sequencing objects from the ST-GCA were obtained as AnnData objects from gutcellatlas.org/. Detailed sample collection and data generation information is available from the original publication (Elmentaite et al., 2021). The Paediatric IBD data (**pIBD**) is made up of 10x Genomics SC-RNAseq data using Chromium Single-Cell 3' gene expression v2 reagents. The count matrix as produced by cell-ranger v.2.1.1 in the original publication was used and is available from GEO (GSE169136). The complete metadata was obtained from the repository https://github.com/ramvinay/Kokkinou_pIBD. Detailed sample and clinical information is available from the original publication (Kokkinou et al., 2023). The ILC subset pre-processed scRNA-seq data, from the adalimumab IBD dataset (**adaIBD**), were downloaded from https://zenodo.org/records/14007626, details of which can be found in the original publication (Thomas et al., 2024).

**Detailed quality control and processing of the ST-GCA and pIBD datasets**

**ST-GCA:** Data from the space-time gut cell atlas was subset from the original AnnData object to include only cells which originated from the small intestine of organ donors. There was a limited recovery of ILCs from 10X experiments performed on samples of the small intestine and a subset was created for comparison of cells recovered from experiments performed on samples of the Large Intestine. These small (SI) and Large intestine (LI) subsets contained 30 and 162 ILCs respectively before further QC filtering. AnnData objects were converted for further processing to Seurat Objects built under Seurat version 5.1.0 (Hao et al., 2024). The raw count data was split into individual 10X runs for QC and each run was assessed for cell counts and cell type representation. 10X runs in which less than 100 cells were recovered were flagged and QC metrics manually inspected to determine if they should be included in

downstream analysis. All experiments were determined to contain valid cells of suitable quality, and no experiments were thrown out. In the original data, cells for each 10X experiment were filtered for more than 500 features, less than 50% mitochondrial reads and feature expression in more than three cells. Since representation of mitochondrial genes is cell type specific, and 50% mitochondrial reads is too loose a constraint for most immune cell types, each experiment was further subdivided by cell type and cell types were individually restricted by percent mitochondrial reads. Cell types of the small intestine were limited to 20% mitochondrial reads unless the median upper bound for outliers of a given cell type was greater than 20% across all experiments The upper bound for outliers was defined for each cell type in an experiment using the IQR method and the median found across all experiments.

$$Upper\ bound = Q3 + (1.5 \times IQR)$$

For cell types whose median upper bound for outliers exceeded 20%, the default threshold of 50% was retained. The thresholds for mitochondrial reads in the large intestine were set manually based on individual distributions of mitochondrial reads for each cell type. Similar to the small intestine the majority of cell types were capped to 20% or 50% dependant on Q3, but 'LEC3 (ADGRG3+)', 'LEC5 (CLDN11+)', 'Th17', 'Mesothelium(PRG4+)', 'cDC1', 'Macrophages', 'Stem cells', 'gdT', and 'cDC2' cells were capped to 30% mitochondrial reads, and 'IgA plasma cell', 'Activated CD4 T', 'Adult Glia', 'Stromal 1 (ADAMDEC1+)', and 'TA' cells were thresholder to 15%. In all cases, the final threshold for percent mitochondrial reads was more stringent than the original data and served to exclude cells in which mitochondrial genes were excessively represented. Total counts in both LI and SI datasets were limited to 20000 which served primarily to filter out plasma cells and cycling B cells with an over-represented number of transcripts. To account for trends in the data that could be driven by variance, cell types for both LI and SI datasets were downsampled. All cell types in the SI dataset were downsampled to 50 cells unless there were fewer than 50 cells total, in which case

all cell of that type were retained. All cell types in the LI dataset were downsampled to 152 cells, again retaining all cells in cases where there were fewer than 152 total cells. After QC, 28 ILCs were included in the SI dataset and 152 ILCs were included in the LI data set. Finally, cell IDs for the quality controlled and down-sampled data were recorded and the normalised RNA-sequencing data from gutcellatlas.org/ was used for these cell IDs to generate the downstream figures.

**pIBD:** The Paediatric IBD dataset was QC filtered according to the original publication parameters of less than 5% mitochondrial genes, between 200 and 2500 total detected genes, with each gene present in at least three cells. Experiment based batch effects were removed from the data using 'comBat' under the 'sva' package version 3.52.0. The Cell annotations provided were checked for consistency with the original publication by UMAP embedding. Cluster based annotations were found to be consistent using Harmony version 1.2.1 (Korsunsky et al., 2019) for integration of the dimensionally reduced data.

**adaIBD**: The counts expression matrix and metadata loaded into R (v4.4.1) and further processed using Seurat (v5.0.0). No additional filtering steps were performed. For comparison of the expression of *TGFB1* genes, raw counts were pseudobulked using the AggregateExpression function based on the grouping factors: "Patient", "Disease", "Treatment", and "Inflammation". Healthy samples removed for further analysis and additional filtering was performed to remove genes that were not expressed in any samples. Differential expression analysis was performed on the pseudobulked data using DESeq2 (v1.44.0) with the following design formula: ~ patient + inflammation + inflammation:treatment. Genes were considered to be significantly differentially expressed with an adjusted p-value $< 0.05$. For the visualisation of gene expression, DESeq2 normalised values were used.

**Data Visualisation**

**Fig. 4A,B** heatmaps were produced using pheatmap version 1.0.12. The Average expression for each cell type was computed from the log normalised data provided by the gutcellatlas.org/. The average expression for each gene (row) was then mean centre scaled across cell types to best compare relative expression of a given gene between cell types. The **Supplementary Fig. 5B** was produced from a subset of the pIBD data containing only the ILC3 cell type. The aggregated ComBat integrated count data from inflamed and non-inflamed groups is compared as average expression. For visualisation of the expression of prototypical ILC marker genes (**Supplementary Fig. 5**), the data were log transformed and scaled using the standard parameters of the NormalizeData function.

**Analysis of bulk RNA-seq datasets**

Raw sequencing reads (fastq) for the D7 non-irradiated organoids (vehicle vs TGF-β1) were downloaded from GSE222505 and aligned to mouse (mm9) genomes using HISAT2 (v2.2.1) with default parameters. The resultant SAM files were converted to BAM format, sorted, and indexed using samtools (v1.17). Next, gene-level quantification was performed using featureCounts from the Subread package (v2.0.6). Finally, differential gene expression was performed using the DESeq2 package in R, following the removal of lowly expressed genes (fewer than 10 counts across all samples). Genes with an adjusted p-value $<=$ 0.05 were considered significant.

**Statistical analysis**

All data was presented as mean ± SD. For comparison between two independent groups an unpaired, two-tailed t-test was employed. For comparisons of more than 2 independent groups, a one-way analysis of variance followed by Tukey's or Dunnett's multiple comparison test was used. An alpha value of 0.05 was used for all statistical tests.

## SUPPLEMENTAL MATERIALS

## Table 1 Composition of the buffers used

| Buffer | Reagent | Concentration | Supplier |
|---|---|---|---|
| **PBS2** | PBS (1 X) 7.2 pH | 1 X | ThermoFisher Scientific |
| | Foetal Bovine Serum | 2% | Gibco |
| **PBS10** | PBS (1 X) 7.2 pH | 1 X | Gibco |
| | Foetal Bovine Serum | 10% | Gibco |
| **FACS buffer** | PBS2 | 1 X | - |
| | HEPES (1M) | 2 mM | ThermoFisher Scientific |
| | Ethylenediamine Tetraacetic Acid | 1 mM | ThermoFisher Scientific |
| **Epithelium removal buffer** | PBS (1 X) 7.2 pH | 1 X | ThermoFisher Scientific |
| | HEPES 1M | 10 mM | ThermoFisher Scientific |
| | Ethylenediamine Tetraacetic Acid | 5 mM | ThermoFisher Scientific |
| **Digestion buffer** | PBS (1 X) 7.2 pH | 1 X | ThermoFisher Scientific |
| | Foetal Bovine Serum | 1% | Gibco |
| | Collagenase | 500 µg/mL | Merck |
| | Dispase | 1.5 mg/mL | Merck |
| | DNase1 | 500 µg/mL | Merck |
| **Isotonic Percoll** | Percoll | 90% | Cytiva |
| | Hanks' Balanced Salt Solution (10X) | 10% | Gibco |
| **Crypt isolation buffer** | PBS (1 X) 7.2 pH | 1 X | Thermo Fisher Scientific |
| | Ethylenediamine Tetraacetate Acid | 5 mM | Thermo Fisher Scientific |
| **MACS buffer** | PBS (1 X) 7.2 pH | 1 X | Thermo Fisher Scientific |
| | Ethylenediamine Tetraacetic Acid | 2 mM | Thermo Fisher Scientific |
| | Bovine Serum Albumin | 0.50% | Sigma-Aldrich |
| **Staining buffer** | PBS (1 X) 7.2 pH | 1 X | Thermo Fisher Scientific |
| | Triton-X-100 | 0.20% | Sigma-Aldrich |
| | Sodium azide | 0.05% | Sigma-Aldrich |
| **Blocking solution** | Staining buffer | 1 X | - |
| | Foetal bovine serum | 5% | Gibco |
| **Washing buffer** | Staining buffer | 1 X | - |
| | Tween-20 | 0.10% | Sigma-Aldrich |
| **MACS buffer** | PBS (1 X) 7.2 pH | 1 X | Thermo Fisher Scientific |
| | Ethylenediamine Tetraacetic Acid | 2 mM | Thermo Fisher Scientific |
| | Bovine Serum Albumin | 0.50% | Sigma-Aldrich |
| **Chelation buffer** | Distilled water | 1 X | Thermo Fisher Scientific |
| | $Na_2HPO_4$ | 5.6 mM | Sigma-Aldrich |
| | $KH_2PO_4$ | 8.0 mM | Sigma-Aldrich |
| | NaCl | 96.2 mM | Sigma-Aldrich |
| | KCl | 1.6 mM | Sigma-Aldrich |
| | Sucrose | 43.4 mM | Sigma-Aldrich |
| | d-sorbitol | 54.9 mM | Sigma-Aldrich |
| | dl-dithiothreitol | 1 M | Sigma-Aldrich |
| | Ethylenediamine Tetraacetic Acid | 0.5 M | Thermo Fisher Scientific |

| | | | |
|---|---|---|---|
| **Neutralisation buffer** | Dulbecco's Modified Eagle Medium Nutrient Mixture F-12 (Ham) | 1 X | Gibco |
| | Foetal Bovine Serum | 10% | Gibco |

## Table 2 Composition of different media used

| Medium | Reagent | Concentration | Supplier |
|---|---|---|---|
| **Basal medium** | Dulbecco's Modified Eagle Medium Nutrient Mixture F-12 (Ham) | - | Gibco |
| | GlutaMAX (200mM) | 2mM | Gibco |
| | HEPES (1M) | 10mM | Corning |
| | Antimycotic-antibiotic (100X) | 1X | Gibco |
| | N2 supplement (100X) | 1X | Gibco |
| | B27 supplement (50X) | 1X | Gibco |
| | N-acetylcysteine (500mM) | 1mM | Sigma |
| **ENR medium** | Basal medium | 1 X | - |
| | Noggin supernatant | 2% | Supernatant from HEK293 cells expressing R-spondin or Noggin |
| | R-spondin supernatant | 2% | |
| | EGF | 50 ng/µL | R&D Systems |
| **Murine co-culture medium** | ENR medium | 1X | - |
| | rhIL-2 | 20 ng/mL | BioLegend |
| | r mL-7 | 20 ng/mL | BioLegend |
| **Complete DMEM medium** | DMEM | 1 X | Gibco |
| | Foetal Bovine Serum | 10% | Gibco |
| | GlutaMAX (200mM) | 2mM | Gibco |
| | Penicillin/Streptomycin | 100 µg/mL | Gibco |
| | HEPES (1M) | 10mM | Corning |
| | β-mercaptoethanol | 20 µM | R&D Systems |
| | rhIL-2 | 20 ng/mL | BioLegend |
| | r mL-7 | 20 ng/mL | BioLegend |
| **TMLC medium** | High glucose DMEM | 1 X | Gibco |
| | Penicillin/Streptomycin | 1% | Gibco |
| | Foetal Bovine Serum | 10% | Gibco |
| **Complete organoid growth medium** | IntestiCult Organoid Growth Medium (human) | 1 X | StemCell |
| | Primocin | 2% | InvivoGen |
| | Y-27632 dihydrochloride | 10 µM | StemCell |
| **Complete organoid differentiation medium** | IntestiCult Organoid Differentiation Medium (human) | 1 X | StemCell |
| | DAPT | 5 mM | Sigma-Aldrich |
| | Primocin | 2% | InvivoGen |
| | Complete organoid differentiation medium | 50% | StemCell |

| | | | |
|---|---|---|---|
| **Human co-culture medium (biopsy derived organoids)** | ENR medium | 50% | - |
| | β-mercaptoethanol | 20 µM | R&D Systems |
| | rhIL-2 | 20 ng/mL | BioLegend |
| | rmL-7 | 20 ng/mL | R&D Systems |

## Table 3 Murine and human flow cytometry antibodies used

| Name | Species | Fluorophore | Clone | Company | Catalogue number |
|---|---|---|---|---|---|
| CD25 | mouse | AlexaFluor488 | eBio3C7 | eBioscience™ | 53-0253-82 |
| CD4 | mouse | AlexaFluor700 | RM4-5 | BioLegend | 557956 |
| CD62L | mouse | APC | MEL-14 | BioLegend | 104412 |
| CD127 | mouse | APC | A7R34 | eBioscience™ | 17-1271-82 |
| CD140a | mouse | APC | APA5 | BioLegend | 135907 |
| IL-22 | mouse | APC | IL22IOP | eBioscience | 17-7222-80 |
| CD4 | mouse | APC Fluor 780 | GK1.5 | eBioscience™ | 47-0041-82 |
| CD326 (EpCAM) | mouse | APC-Cy7 | G8.8 | BioLegend | 118217 |
| CD45 | mouse | BV510 | 30-F11 | BioLegend | 103137 |
| NK1.1 | mouse | BV605 | PK136 | BioLegend | 108753 |
| NKp46 | mouse | BV605 | 29A1.4 | BioLegend | 137619 |
| T-bet | mouse | BV711 | 4B10 | BioLegend | 644819 |
| CD25 | mouse | BV786 | PC61 | BioLegend | 564023 |
| RORγt | mouse | BV786 | Q31-378 | BD Horizon™ | 564723 |
| CD3 | mouse | eFluor450 | 145-2C11 | eBioscience™ | 48-0031-82 |
| CD5 | mouse | eFluor450 | 53-7.3 | eBioscience™ | 48-0051-82 |
| CD19 | mouse | eFluor450 | eBio1D3 | eBioscience™ | 48-0193-82 |
| Ly6G | mouse | eFluor450 | RB6-8C5 | eBioscience™ | 48-5931-82 |
| TER-119 | mouse | eFluor450 | TER-119 | eBioscience™ | 48-5921-82 |
| ITGB3 | mouse | FITC | 2C9.G2 | BioLegend | 104305 |
| CD44 | mouse | PE | IM7 | eBioscience™ | 12-0441-81 |
| ITGAV | mouse | PE | RMV-7 | BioLegend | 104105 |
| Isotype control | mouse | PE | MOPC-21 | BioLegend | 981804 |
| LAP | mouse | PE | TW-20B9 | BioLegend | 141305 |
| NK1.1 | mouse | PE | PK136 | eBioscience™ | 12-5941-83 |
| SMAD-2/3 | mouse | PE | O72-670 | BD Horizon™ | 562586 |
| CD44 | mouse | Pe-Cy7 | IM7 | BioLegend | 10303 |
| CCR6 | mouse | Pe-Cy7 | 29-2L17 | BioLegend | 129816 |
| Foxp3 | mouse | Pe-Cy7 | FJK-16s | eBioscience™ | 25-5773-82 |
| NK1.1 | mouse | Pe-Cy7 | PK136 | eBioscience™ | 25-5941-82 |

| | | | | | |
|---|---|---|---|---|---|
| NKp46 | mouse | Pe-Cy7 | 29A1.4 | eBioscience™ | 25-3351-82 |
| IL-17a | mouse | PE/Dazzle | TC11-18H10.1 | BioLegend | 506937 |
| IFNγ | mouse | PerCPeFluor710 | XMG1.2 | eBioscience™ | 45-7311-80 |
| KLRG1 | mouse | PerCPeFluor710 | 2F1 | eBioscience™ | 46-5893-82 |
| CD56 | human | AlexaFluor700 | HCD56 | BioLegend | 318316 |
| CD4 | human | APC | RPA-T4 | BioLegend | 300514 |
| CD161 | human | APC | HP-3G10 | BioLegend | 339912 |
| ITGB1 | human | APC | TS2/16 | BioLegend | 303007 |
| ITGB8 | human | APC | 416922 | Invitrogen | MA5-23674 |
| ITGB8 | human | Unconjugated | ADWA16 | - | - |
| KLRG1 | human | APC | 14C2A07 | BioLegend | 368606 |
| NKp46 | human | APC | 9E2 | BioLegend | 331918 |
| CD326 (EpCAM) | human | APC-Cy7 | 9C4 | BioLegend | 324246 |
| CD62L | human | BV421 | DREG-56 | BioLegend | 304827 |
| CD45 | human | BV510 | 2D1 | BioLegend | 368526 |
| CD45RA | human | BV510 | HO100 | BioLegend | 304141 |
| CD3 | human | BV605 | OKT3 | BioLegend | 317321 |
| CD117 (cKit) | human | BV605 | 104D2 | BioLegend | 313218 |
| CD25 | human | BV711 | BC96 | BioLegend | 302636 |
| Lineage cocktail (lin 3) | human | FITC | - | Becton Dickinson UK Ltd | 643510 |
| TCR-αβ | human | FITC | IP26 | eBioscience™ | 11-9986-42 |
| TCR-γδ | human | FITC | B1 | BioLegend | 331208 |
| CD25 | human | PE | BC96 | eBioscience™ | 12-0259-80 |
| CD294 (CRTh2) | human | PE | BM16 | BioLegend | 350106 |
| ITGAV (CD51) | human | PE | NKI-M9 | BioLegend | 327909 |
| CD127 | human | PE-Cy7 | eBioRDR5 | Fisher Scientific UK | 25-1278-42 |
| ITGB3 (CD61) | human | PE/Dazzle | VI-PL2 | BioLegend | 336425 |
| CCR6 | human | PE/Dazzle | G034E3 | BioLegend | 353429 |
| NKp44 | human | PerCP-Cy5.5 | P44-8 | BioLegend | 325114 |

## Table 4 Cytokine stimulation of murine NCR$^+$ILC3s and NCR$^-$ ILC3s.

| ILC3s | Media composition | Concentration | Supplier | Catalogue number |
|---|---|---|---|---|
| **NCR$^+$ ILC3s** | Complete DMEM medium | 1 X | - | - |
| | rm IL-12 | 20 ng/mL | Miltenyi | 130-096-707 |
| | rm IL-18 | 20 ng/mL | BioLegend | 767002 |
| **NCR$^-$ ILC3s** | Complete DMEM medium | 1 X | - | - |
| | rm IL-1β | 20 ng/mL | Cambridge Biosciences | RP0359M-005 |
| | rm IL-23 | 20 ng/mL | Miltenyi | 130-096-676 |

**Table 5 List of murine and human TAQ probes used**

| Gene name - mouse | Probe identifier | Supplier |
|---|---|---|
| *Itgav* | Mm00434486_m1 | Thermo Fisher Scientific |
| *Itgb3* | Mm00443980_m1 | |
| *Mmp2* | Mm00439498_m1 | |
| *Mmp9* | Mm00442991_m1 | |
| *Mmp14* | Mm00485054_m1 | |
| *Mmp3* | Mm00440295-m1 | |
| *Tgfbr1* | Mm00436964_m1 | |
| *Tgfbr2* | Mm00436976_m1 | |
| *Tgfb1* | Mm01178820_m1 | |
| *Vim* | Mm01333430_m1 | |
| *Clu* | Mm01197002_m1 | |
| *Yap1* | Mm01143263_m1 | |
| *Il22* | Mm01226722_g1 | |
| *Ifng* | Mm01168134_m1 | |
| **Gene name - human** | **Probe identifier** | **Supplier** |
| *IL22* | Hs01574154_m1 | Thermo Fisher Scientific |
| *IL17A* | Hs00174383_m1 | |
| *TGFB1* | Hs00998133_m1 | |
| *TGFBR1* | Hs00610320_m1 | |
| *TGFBR2* | Hs00234253_m1 | |
| *MUC2* | Hs03005103_g1 | |
| *ITGAV* | Hs00233808_m1 | |
| *MMP14* | Hs01037003_g1 | |
| *GAPDH* | Hs02786624_g1 | |

**Table 6 List of human primers used**

| Gene name | Forward primer sequence | Reverse primer sequence |
|---|---|---|
| *LYZ* | TCAATAGCCGCTACTGGTGTA | ATCACGGACAACCCTCTTTGC |
| *MKI67* | CGTCCCAGTGGAAGAGTTGT | CGACCCCGCTCCTTTTGATA |
| *GAPDH* | ACCCACTCCTCCACCTTTGA | CTGTTGCTGTAGCCAAATTCGT |

**Table 7 Antibodies used for immunostaining**

| Antibody | Catalogue number | Supplier |
|---|---|---|
| MUC2 | MA5-12345 | Thermo Fisher Scientific |
| CD45 | 304001 | BioLegend |
| E-cadherin | AF748 | Biotechne |
| Phalloidin (dye) | A30107 | Life Technologies |
| Hoechst 33342 Solution (dye) | 62249 | Thermo Fisher Scientific |
| Alexa Fluor 488 | A27034 | Thermo Fisher Scientific |
| Alexa Fluor 488 | A11001 | Thermo Fisher Scientific |
| Alexa Fluor 568 | A11057 | Life Technologies |
| Alexa Fluor 568 | A10037 | Life Technologies |

**Table 8 Software for scRNA sequencing data analysis**

| Software | Additional information | |
|---|---|---|
| Python 3.12.5 | The Python Software Foundation | https://www.python.org/ |
| R v.4.4.1 | R Core Team | https://cran.r-project.org/ |
| harmony_1.2.1 | Korsunsky et al., 2019 | https://github.com/immunogenomics/harmony |
| zellkonverter_1.14.1 | Zappia et al., 2024 | https://doi.org/doi:10.18129/B9.bioc.zellkonverter |
| Seurat_5.1.0 | Hao et al., 2024 | https://satijalab.org/seurat/ |
| pheatmap_1.0.12 | Kolde R (2018) | https://doi.org/10.32614/CRAN.package.pheatmap |
| sva_3.52.0 | Leek et al., 2024 | https://doi.org/doi:10.18129/B9.bioc.sva |
| SingleCellExperiment_1.26.0 | Amezquita et al., 2020 | https://doi.org/doi:10.18129/B9.bioc.SingleCellExperiment |
