# Supplementary material for: ILC3s mediate intestinal immune-epithelial interactions via TGF-β1 activation
Source: Mucosal Immunol. 2026 Apr;19(2):1735–48. doi: 10.1016/j.mucimm.2025.11.013 (PMC13195368; doi:10.1016/j.mucimm.2025.11.013)
Supplement: Supplementary Data 2 [file mmc2.pdf]

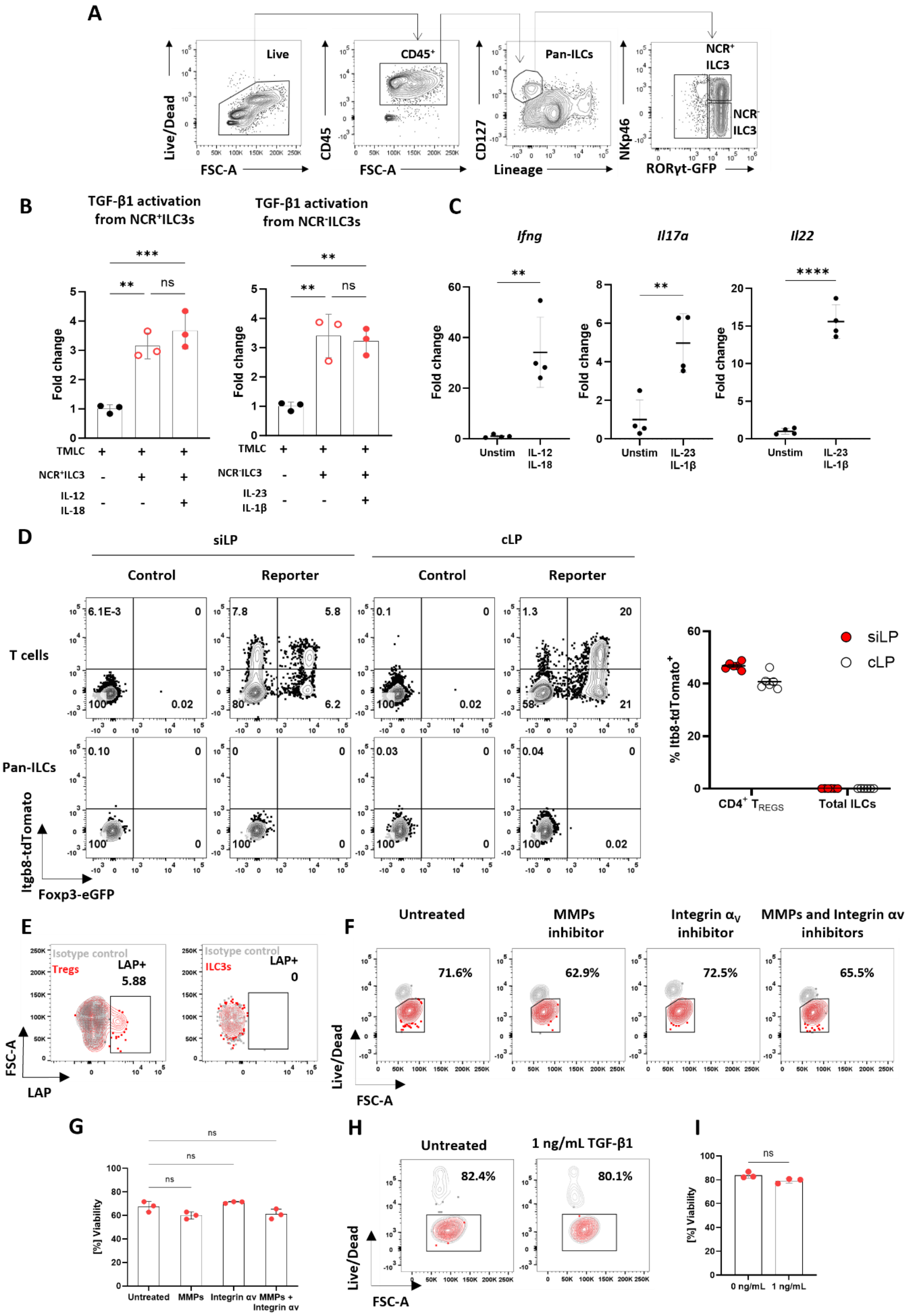
A
Live
CD45+
Pan-ILCs
NCR+ ILC3
NCR- ILC3
Live/Dead
FSC-A
CD45
CD127
Lineage
NKp46
RORγt-GFP
B
TGF-β1 activation from NCR+ILC3s
TGF-β1 activation from NCR-ILC3s
Fold change
TMLC
NCR+ILC3
IL-12 IL-18
NCR-ILC3
IL-23 IL-1β
C
Ifng
Il17a
Il22
Unstim
IL-12 IL-18
IL-23 IL-1β
D
siLP
cLP
Control
Reporter
T cells
Pan-ILCs
Itgb8-tdTomato
Foxp3-eGFP
% Itgb8-tdTomato+
siLP
cLP
CD4+ TREGS
Total ILCs
E
Isotype control
Tregs
ILC3s
LAP+
5.88
FSC-A
LAP
F
Untreated
MMPs inhibitor
Integrin αV inhibitor
MMPs and Integrin αv inhibitors
71.6%
62.9%
72.5%
65.5%
Live/Dead
FSC-A
G
[%] Viability
Untreated
MMPs
Integrin αv
MMPs + Integrin αv
H
Untreated
1 ng/mL TGF-β1
82.4%
80.1%
I
0 ng/mL
1 ng/mL

Supplementary Figure 1. Small intestinal ILC3s produce and activate TGF-β1. (A) Representative flow cytometric plots illustrating the gating strategy used to isolate siLP ILC3s. (**B**) Active levels of TGF-β1 detected in co-cultures of TMLC with unstimulated or stimulated NCR^{+} ILC3s, followed by the levels of active TGF-β1 detected in co-cultures of TMLC with unstimulated or stimulated NCR^{-} ILC3s, *n*=3 (**C**) *Ifng*, *Il17a* and *Il22* gene expression detected from unstimulated or stimulated ILC3s with pro-inflammatory cytokines, *n*=4. (**D**) Representative flow cytometric plots illustrating the expression of Itgb8-tdTomato from FoxP3-$eGFP^{+}$ Tregs and from ILCs ($CD45^{+}Lin^{-}CD127^{+}CD90^{+}$), isolated from the small intestinal lamina propria (siLP) and colonic lamina propria (cLP) of dual FoxP3-eGFP/Itgb8-tdTomato reporter mice followed by their quantification, *n=6.* (**E**) Representative flow cytometry histograms illustrating the expression of LAP on the surface of Tregs vs siLP ILC3s, where the red contour population are the Tregs and siLP ILC3s respectively and the grey contour population is the isotype control. (**F**) Representative flow cytometry histograms displaying the viability of siLP ILC3s in the different treatment conditions, followed by (**G**) the quantification of viable cells, *n*=3. (**H**) Representative flow cytometry histograms indicating the viability of siLP ILC3s in the presence or absence of TGF-β1, followed by (**I**) the quantification of viable cells, *n*=3. Data are represented as mean ±SD. Statistics: (**B**) one-way ANOVA, followed by Tukey's multiple comparison test (**C, I**) unpaired, two-tailed t-test, (**G**) one-way ANOVA, followed by Dunnett's multiple comparison test. (ns, non-significant = $p > 0.05$, ** $p \leq 0.01$, *** $p \leq 0.001$, **** $p \leq 0.0001$).

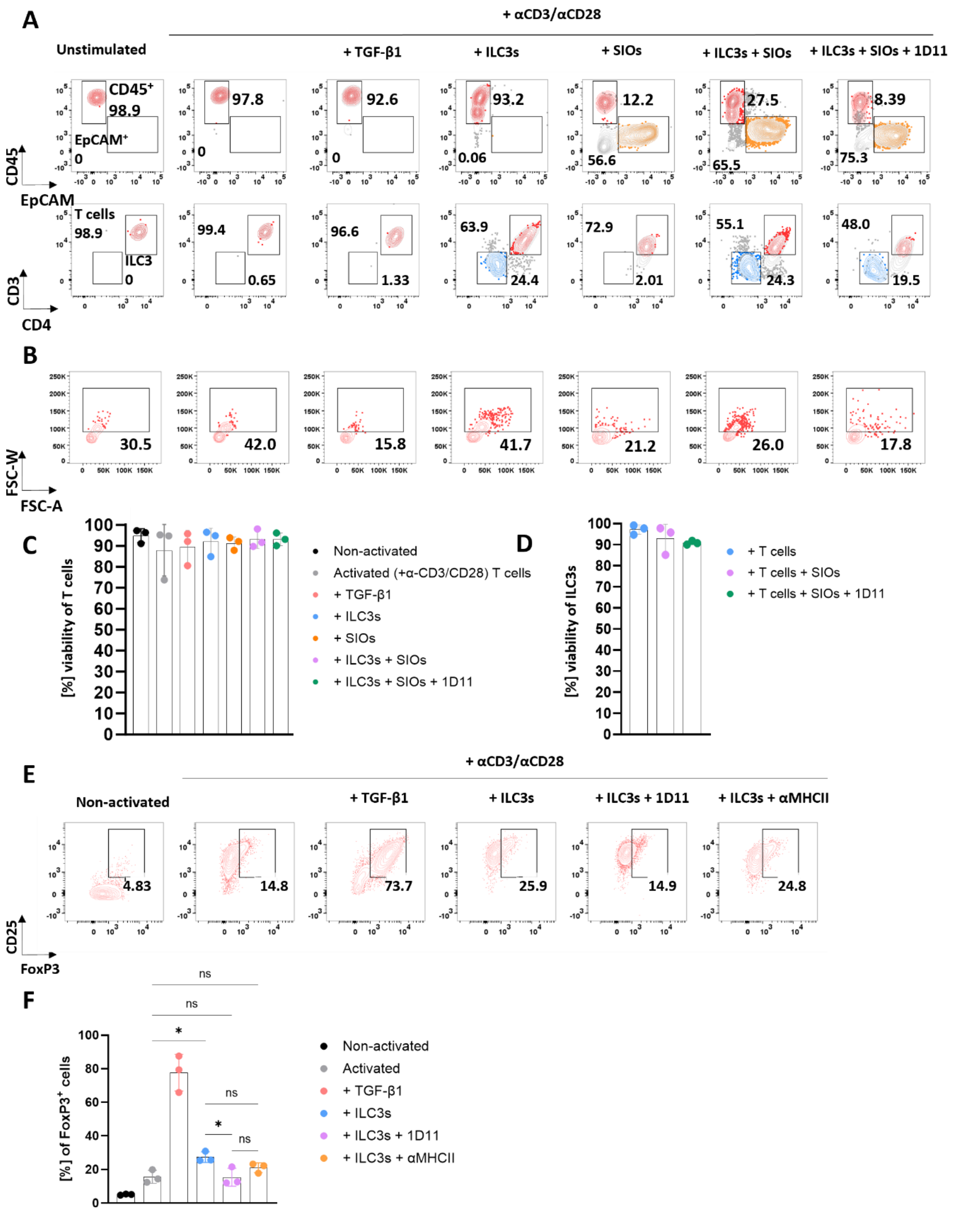

Supplementary Figure 2. Tri-culture characterisation and viability of immune cells. (A) Representative flow cytometric contour plots of the cell populations identified in the conditions based on CD45 and EpCAM expression, illustrating the separation of T cells ($CD45^{high}$ $EpCAM^{-}$), ILC3s ($CD45^{low}$ $EpCAM^{-}$) and epithelial cells ($CD45^{-}$ $EpCAM^{+}$) pre-gated on

single, live cells followed by the representative flow cytometric plots illustrating the separation of ILC3s and T cells based on the expression of CD3 and CD4. (**B**) Representative flow cytometric contour plots of the cell scatter. (**C**) Frequency of live T cells, *n*=3. (**D**) Frequency of live siLP ILC3s, *n*=3. (**E**) Representative flow cytometric contour plots of $CD25^{+}FoxP3^{+}$ T cells (pre-gated on $CD3^{+}CD4^{+}$ T cells) in single culture of T cells or co-cultures with siLP ILC3s on plates pre-coated with anti-CD3/CD28 activating antibodies. (**F**) Frequency of $FoxP3^{+}$ cells *n*=3. Data are represented as mean ± SD. Statistics: (**F**) one-way ANOVA, followed by Tukey's multiple comparison test. (ns, non-significant = $p > 0.05$, * $p \leq 0.05$).

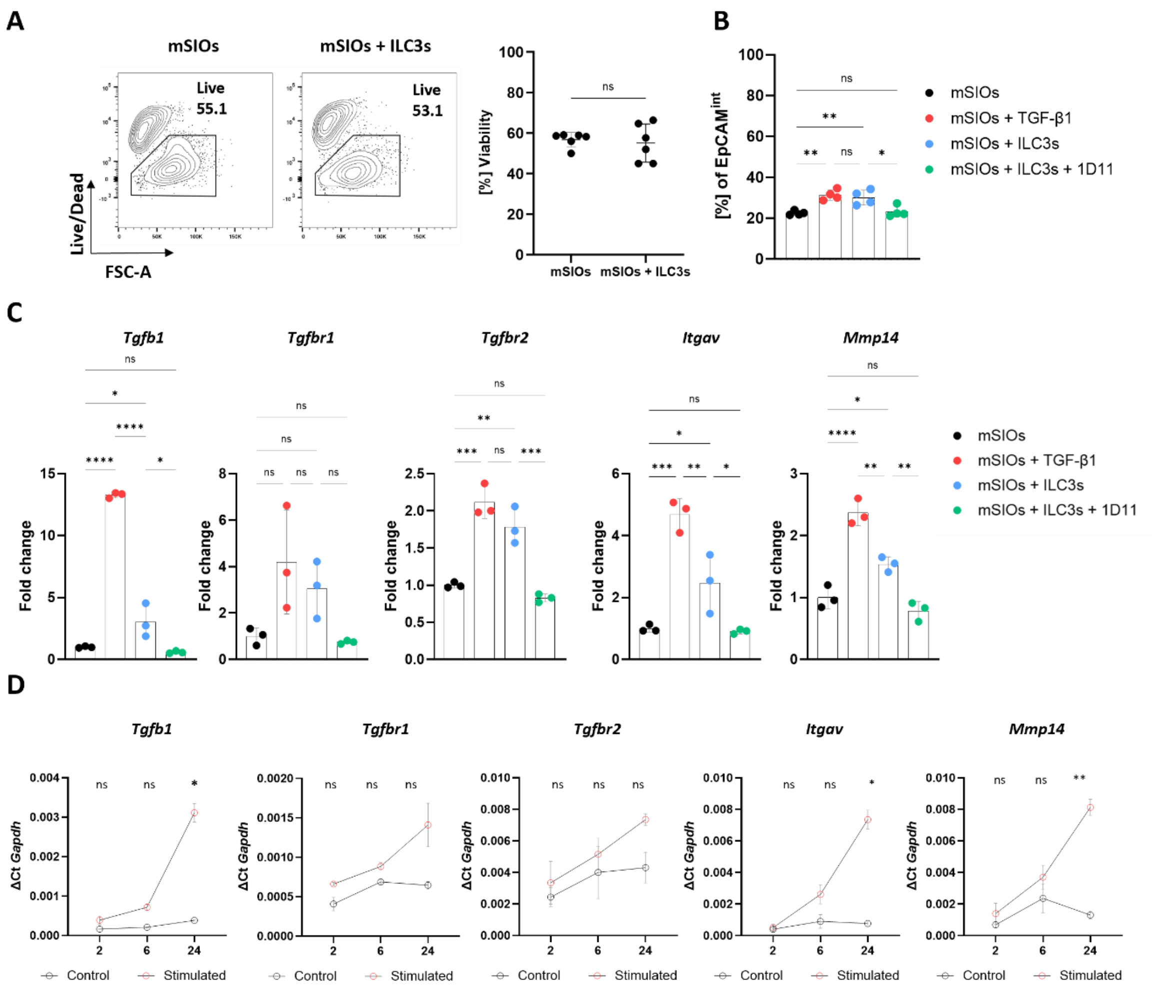

Supplementary Figure 3. mSIOs respond to TGF-β1 treatment and upregulated genes associated with EMT and regeneration. (**A**) Representative flow cytometric contour plots showing the live population of cells in the two conditions, followed by the quantification of viable cells, *n=6*. (**B**) Quantification of the intermediate EpCAM expressing population, *n*=4. (**C**) *Tgfb1, Tgfbr1, Tgfbr2, Itgav* and *Mmp14* expression in IECs isolated from mSIOs cultured under conditions described in (**Fig. 3C**), *n*=3. (**D**) *Tgfb1*, *Tgfbr1, Tgfbr2, Itgav* and *Mmp14* gene expression from mSIOs stimulated for 2, 6 and 24 hours with recombinant TGF-β1, *n*=3 technical replicates for the stimulated condition and *n*=3 technical replicate for the control. Data are represented as mean ±SD. Statistics: (**A**) unpaired, two-tailed t-test, (**B** and **C**) one-way ANOVA followed by Tukey's multiple comparison test and by Tukey's multiple

comparison test respectively, (**D**) two-way ANOVA followed by Šídák's multiple comparisons test. (ns, non-significant = p >0.05, * p≤0.05, ** p ≤0.01, *** p ≤0.001, **** p ≤0.0001).

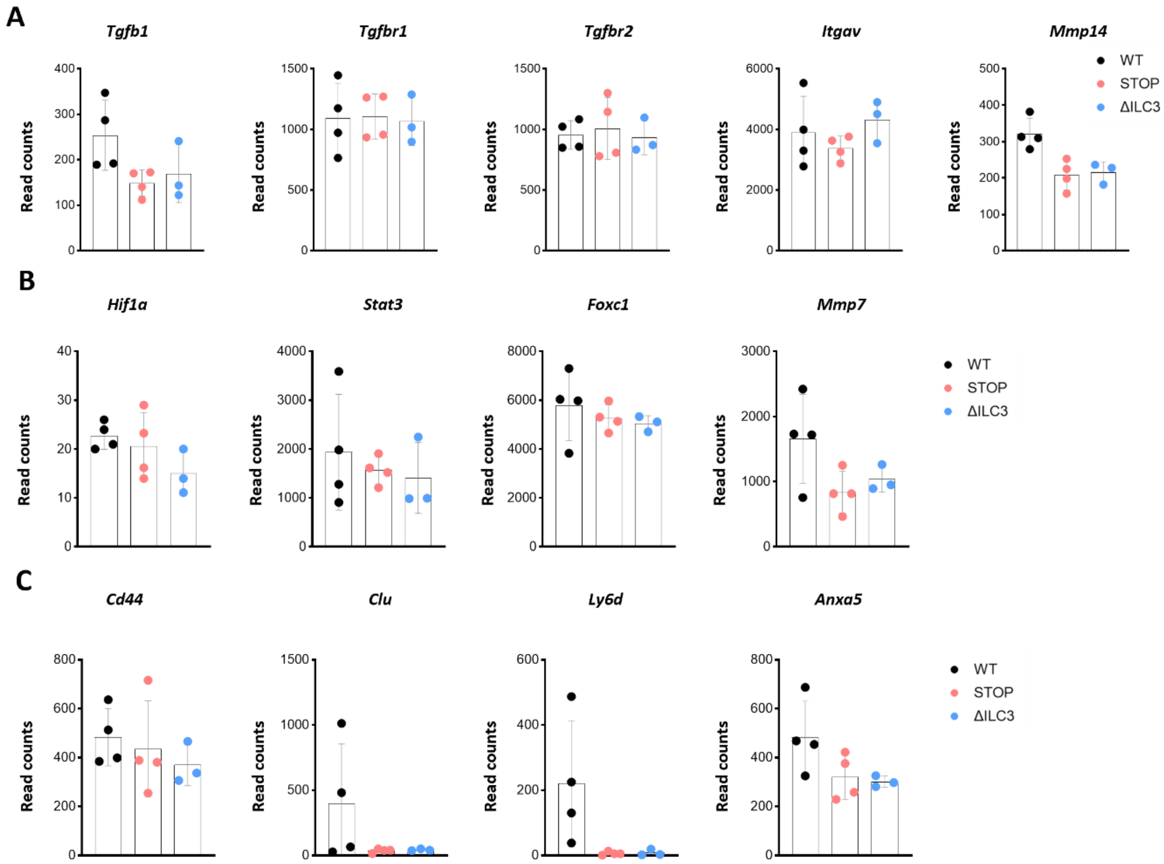

Supplementary Figure 4. Expression of genes associated with TGF-β1 signalling in IEC. (**A**) *Tgfb1, Tgfbr1, Tgfbr2*, *Itgav* and *Mmp14* gene expression from epithelial cells isolated from WT, STOP (lacking both T cells and ILC3s) and ΔILC3 mice (lacking ILC3s only)[46]. (**B**) *Hif1a*, *Stat3*, *Foxc1* and *Mmp7* expression by epithelial cells from WT, STOP and ΔILC3 mice. (**C**) *Cd44*, *Clu*, *Ly6d* and *Anxa5* expression by epithelial cells from WT, STOP and ΔILC3 mice.

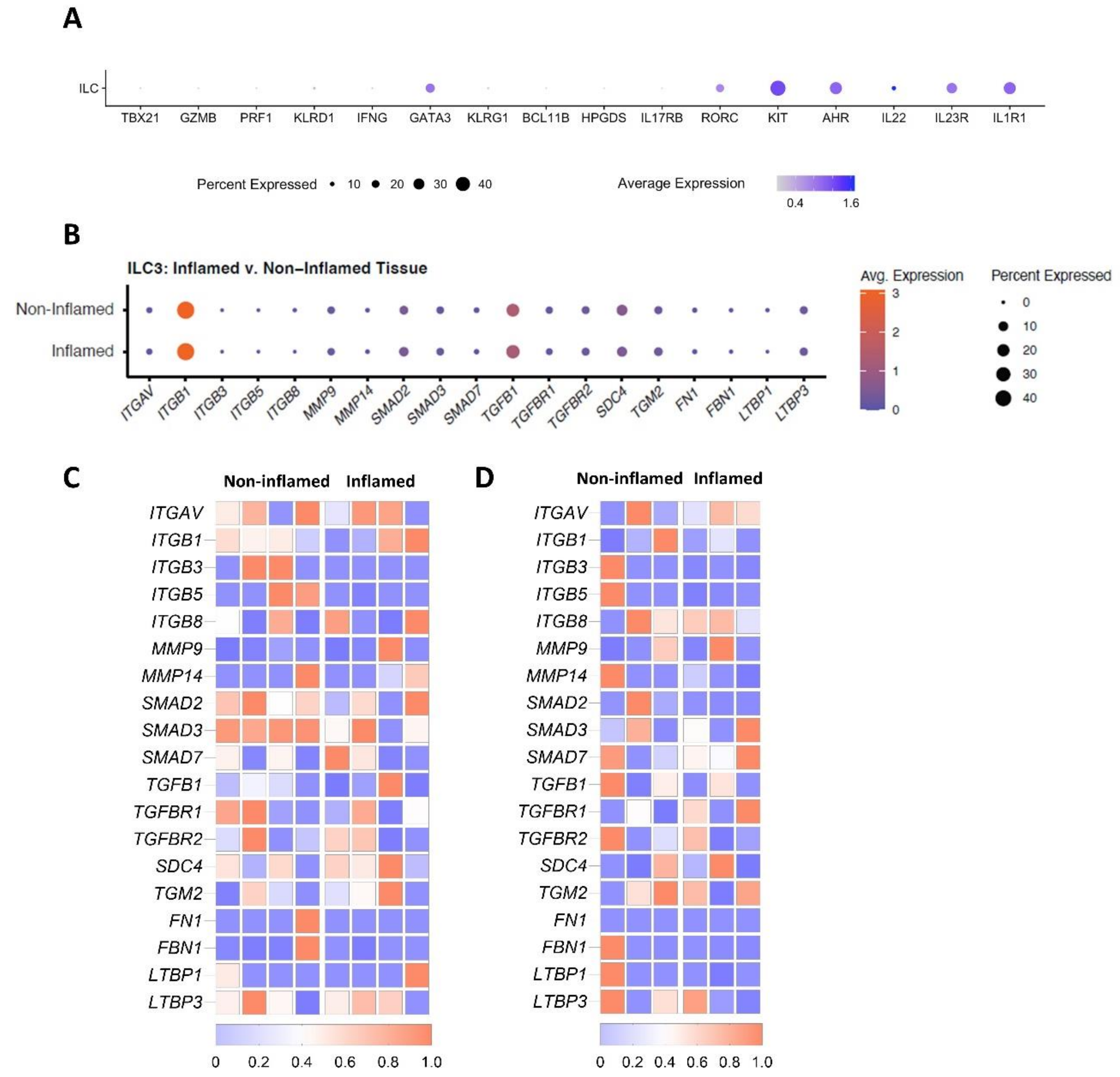

Supplementary Figure 5. TGF-β1 activation and signalling in colonic ILC3s isolated from patients with IBD. (**A**) Dot plot showing the average and percentage expression of prototypical ILC subset marker genes (ILC1: *TBX21*, *GZMB*, *PRF1*, *IFNG*, *KLRD1*; ILC2: *GATA3*, *KLRG1*, *BCL11B*, *HPGDS*, *IL17RB*; ILC3: *RORC*, *KIT*, *AHR*, *IL22*, *IL23R*, *IL1R1*) in scRNA-seq data taken from a publicly available dataset[49]. (**B**) Expression of TGF-β1 signature genes in ILC3 isolated from inflamed and non-inflamed regions of the colon of paediatric individuals with UC (re-analysis of a publicly available dataset[50]). (**C**) Heatmap illustrating the difference in transcript levels between ILC3s isolated from colonic regions with active disease (inflamed) compared to the ILC3s isolated from the adjacent non-inflamed regions in patients

with CD. (**D**) Heatmap illustrating the differences between ILC3s isolated from regions with active disease (inflamed) compared to the ILC3s isolated from the adjacent non-inflamed regions in patients with UC. For both (**C**) and (**D**) ILC3s were gated as gate as live $CD45^{+}Lineage^{-}$ (CD3e, CD34, CD19, CD94, CD14, CD123, FcεRIa, CD11c) $CD127^{+}$, $CRTH2^{-}$, $CD117^{+}$ (re-analysis of a publicly available dataset[51]).

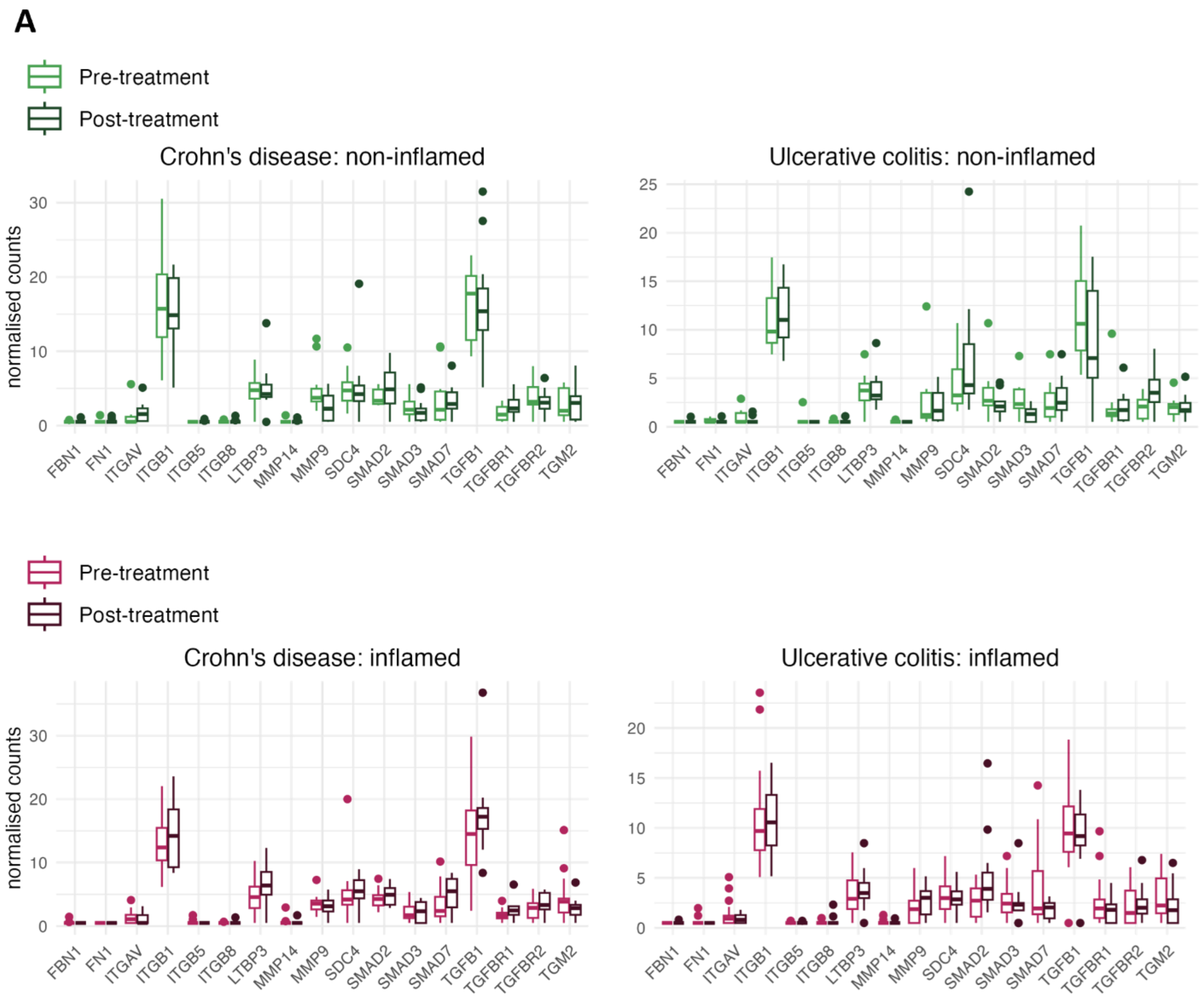

Supplementary Figure 6. TGF-β1 activation and signalling in intestinal ILC3s isolated from patients pre and post treatment with adalimumab. (**A**) TGF-β1 signature genes in ILC3 isolated from inflamed and non-inflamed regions of the colon of individuals with Crohn's Disease or Ulcerative Colitis, either before or after adalimumab (anti-TNF) treatment. Data shown represents normalised counts of pseudobulked scRNA-seq data taken from a publicly available dataset[51].

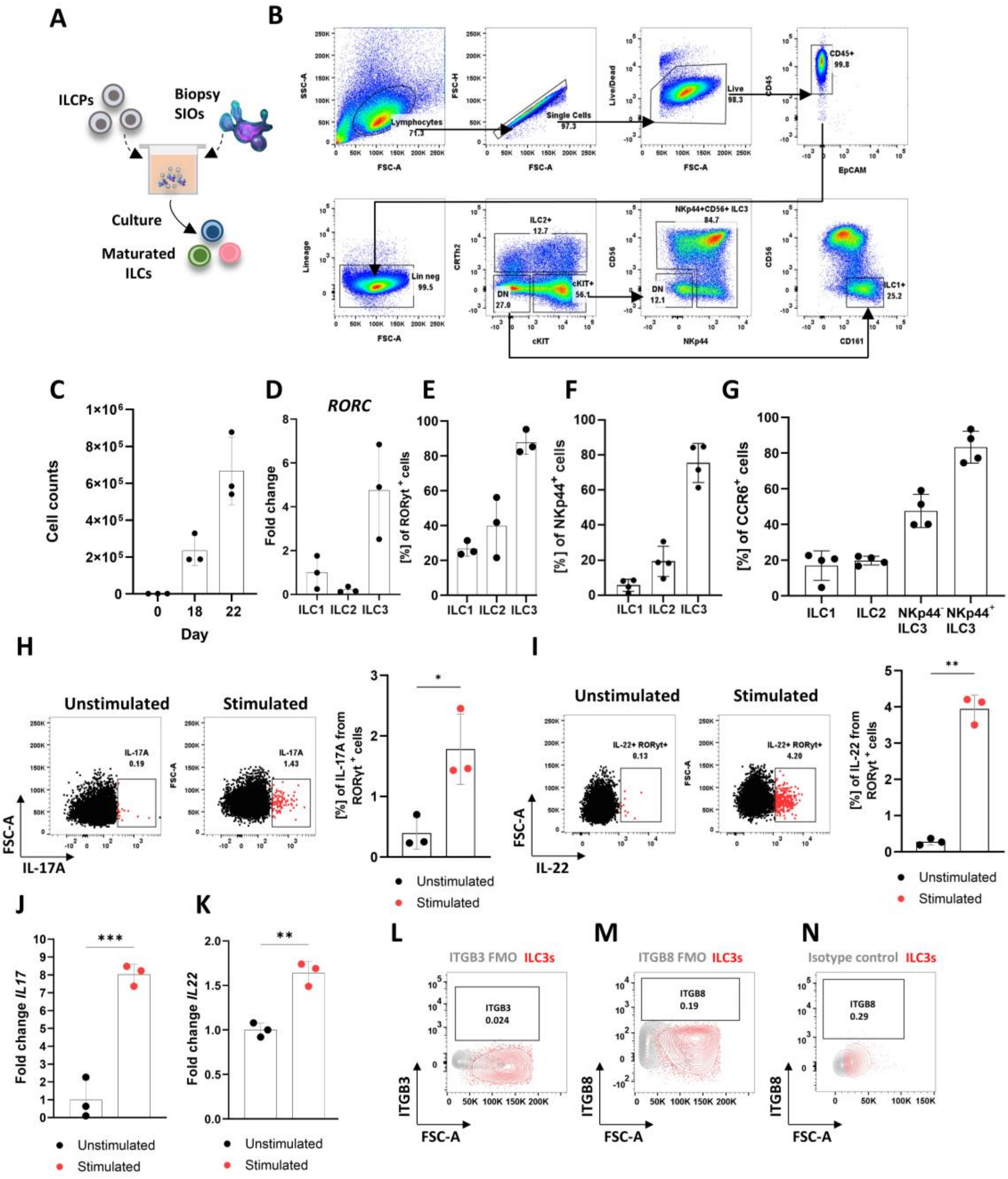

Supplementary Figure 7. Generation and characterisation of hSIO-matured ILC3s. (**A**) Schematic representation of the protocol for generating hSIO-maturated ILC3s. (**B**) hSIO-maturated ILC3s were FACS purified as EpCAM$^-$ CD45$^+$ Lin$^-$ CRTh2$^-$ cKIT$^+$. (**C**) Cell counts of total ILCs expanded after up to 22 days of co-culture. (**D**) *RORC* transcript levels detected from ILC1s, ILC2s and ILC3s. (**E**) Frequency of RORγt$^+$ expressing cells identified in the hSIO-maturated ILCs. (**F**) Frequency of NKp44$^+$ cells quantified from ILC1s, ILC2s and ILC3s. (**G**) Frequency of CCR6$^+$ cells quantified from ILC1s, ILC2s, NKp44$^+$ and NKp44$^-$

ILC3s. (**H**) Representative flow cytometric plots illustrating the ability of ILC3s to produce IL-17 upon stimulation with PMA/Ionomycin, followed by the quantification of $IL17^{+}$ $ROR\gamma t^{+}$ ILC3s , *n=3*. (**I**) Representative flow cytometric plots illustrating the ability of ILC3s to produce IL-22 upon stimulation with PMA/Ionomycin, followed by the quantification of $IL22^{+}$ $ROR\gamma t^{+}$ ILC3s, *n=3*. (**J**) *IL17* transcript level from ILC3s after PMA/Ionomycin stimulation, *n=3*. (**K**) *IL22* transcript level from ILC3s after PMA/Ionomycin stimulation, *n*=3. Representative flow cytometric plot showing the surface expression of ITGB3 (**L**) and ITGB8 (**M**) on hSIO-matured ILC3s, using commercially available antibodies; the red contour populations represent hSIO-matured ILC3s, while grey contour populations correspond to FMOs. (**N**) Representative flow cytometric plot showing the surface expression of ITGB8 using an APC-conjugated anti-integrin β8 antibody (clone ADWA16)[68]. All data are represented as mean ±SD. Statistics: (**H**-**K**) two-tailed t-test. (* $p \leq 0.05$, ** $p \leq 0.01$, *** $p \leq 0.001$).

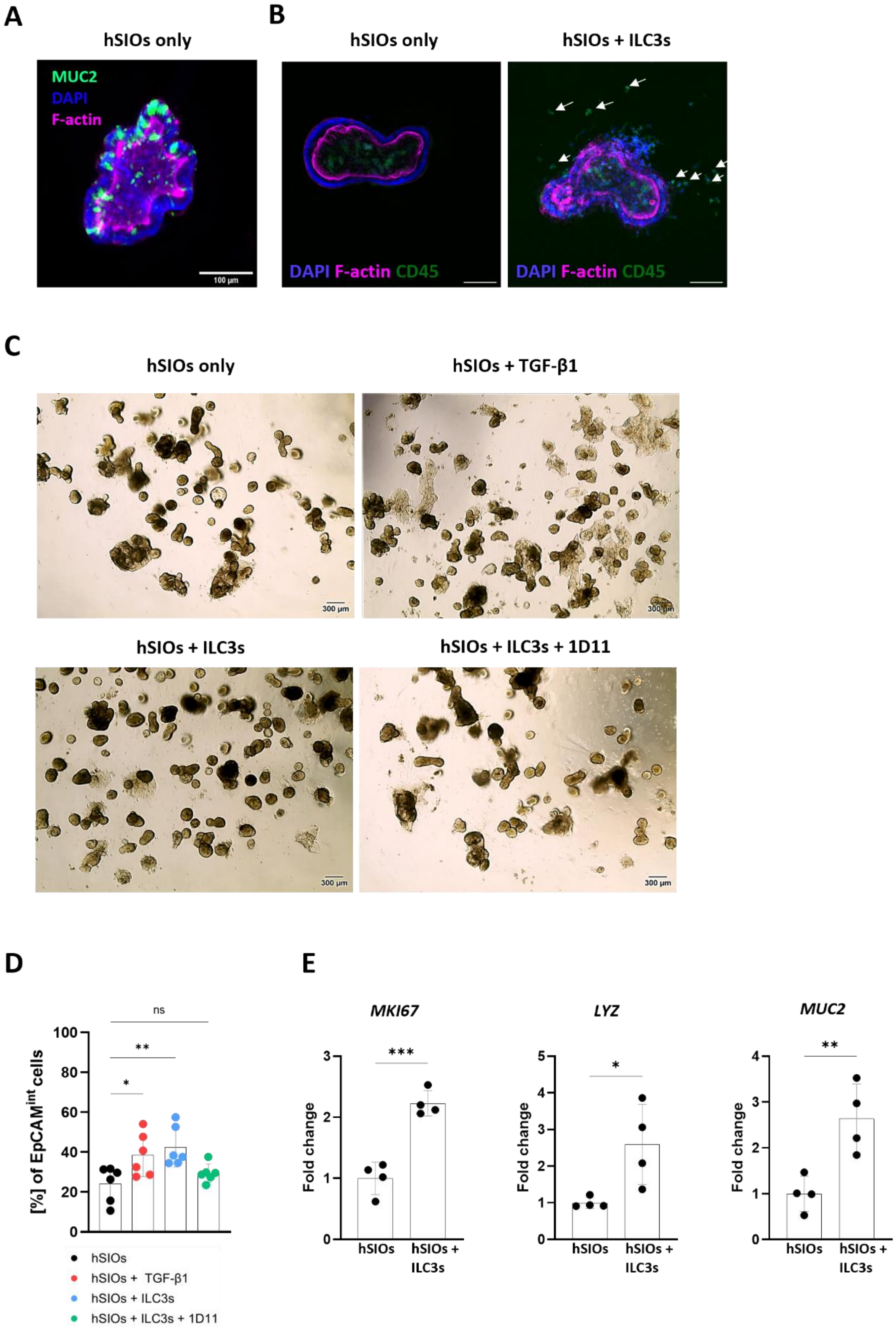

Supplementary Figure 8. Generation and characterisation of hSIO-matured ILC3s. (**A**) Visual representation of maturated hSIOs stained with Phalloidin – F-actin (magenta) stained with DAPI – nucleic acid (blue) and Muc2 – goblet cells (green), scale bar = 100 μM. (**B**)

Visual representation of maturated hSIOs cultured on their own or in co-culture with hSIOs-derived ILC3s (indicated by the white arrows) stained with Phalloidin – F-actin (magenta) stained with DAPI – nucleic acid (blue) and CD45 (green), scale bar = 100 μM. (**C**) Representative images of organoids cultured on their own, in the presence of exogenous TGF-β1, co-cultured with ILC3s or co-cultured with ILC3s and neutralising TGF-β1 antibody (1D11), scale bar = 300 μM. (**D**) Frequency of EpCAM intermediate population quantified from hSIOs cultured in different conditions, n=3. (**E**) *MKI67, LYZ* and *MUC2* transcript expression from hSIOs cultured on their own or in co-culture with hSIOs-derived ILC3s, n=4 All data are represented as mean ±SD. Statistics: (**D**) one-way ANOVA followed by Dunnett's multiple comparison test, (**E**) unpaired, two-tailed t-test. (ns, non-significant = $p > 0.05$, * $p \leq 0.05$, ** $p \leq 0.01$, *** $p \leq 0.001$).

Supplementary Table 1. The differentially expressed genes overlapping between the two datasets analysed and presented in **Fig. 3J**.

Overlapping Differentially Expressed Genes				
Reg3g	*Ghr*	*Brip1*	*Incenp*	*Apobec3*
Reg3b	*Duoxa2*	*Cyp3a11*	*Gstm1*	*Pthlh*
Tifa	*Cyp2c38*	*Ttc39c*	*Gsta3*	*Slc17a4*
Mptx2	*Ugt2a3*	*Ephx1*	*Hk2*	*Akr1c13*
Mmp7	*Cyp2c68*	*Aldh1a1*	*Slc7a7*	*Gstm6*
Hmgcs2	*Aqp11*	*Cdkn1a*	*Hif3a*	*Fbp2*
Pla2g2a	*Akr1c19*	*Mcl1*	*Ugt2b34*	*Cd24a*
Ceacam1	*Cat*	*Aldh1a7*	*Bhlha15*	*Plk2*
Ano1	*Ffar4*	*Hacl1*	*Gstt1*	*Tff3*
Slc6a4	*Mgat4c*	*Cryl1*	*Mgst1*	*Slc30a7*
Hsd17b2	*Defa38*	*Nr3c1*	*Coq8a*	*Pafah2*
Abcb1a	*Zhx1*	*Nupr1*	*Pcyox1*	*Zfp937*
Defa39	*Emc9*	*Elmod3*	*Cyp2c65*	*Cep295*
Ces1g	*Fmo5*	*Gsta1*	*Tmem238l*	*Tcn2*
Gstm4	*Ifi27l2b*	*Cyp4f13*	*Akr1c14*	*Ifngr2*
Ugt1a6a	*Abcg2*	*Btnl1*	*Defa22*	*Fmo4*
Cfi	*Kank2*	*Cd44*	*Pink1*	*Anxa1*
Ctrb1	*Rnase1*	*Tmem86b*	*Rgmb*	*Mgst3*
Ugt2b5	*Acadm*	*Ly6a*	*Ace2*	*Smox*
Ces1f	*Ugt2b36*	*Bdh1*	*Cidec*	*Samd5*
Cyp4f16	*Aoc1*	*Zfyve26*	*Erfe*	*Hal*
Defa21	*Adh4*	*Sult1d1*	*Mtarc2*	*Abcc3*
Cyp2c29	*Plgrkt*	*Ech1*	*Tsc22d3*	*Bphl*
Cbr3	*Fabp2*	*Pla2g5*	*Ice2*	*Thbs1*
Slc27a2	*Ces2c*	*Fam98c*	*Alpi*	*Tmed9*
Pbld2	*Bcl2l15*	*Slc26a3*	*B4galnt1*	*Tgm2*
Fbp1	*Trp53i11*	*Ugt1a1*	*Mcm8*	*Flnb*
Ces1d	*Ces1e*	*Pfkfb3*	*Rdh7*	*Slc6a19*
Arg2	*Ccl9*	*Cym*	*Acy1*	*Atad2*
Cth	*Ces2g*	*Fahd1*	*Gstm3*	*Osbpl6*
Cyp4v3	*B3galt5*	*Btd*	*Gde1*	*Gsta4*
Rgn	*Ang*	*Cyp3a25*	*Mylk*	*Rnf43*
Tubb2b	*Pparg*	*Aldh18a1*	*Sorcs2*	*Zfp97*
Nqo1	*Slc22a18*	*Klk1*	*Acer1*	*Zc3hav1*
Ddc	*Il17rd*	*Enc1*	*Maoa*	*Anpep*
Gcnt3	*S100g*	*Qsox1*	*Acy3*	*Pxmp4*
Habp2	*Ces2a*	*Pdss1*	*Ttc39a*	*Acox2*
Zfp462	*Klf10*	*Fut2*	*Dnaaf1*	*Guca2a*
Defa29	*Lcn2*	*Ccl6*	*Hgfac*	*Hadh*
Spink4	*Etfa*	*Gstm7*	*Amn*	*Gse1*
Treh	*Spns2*	*Clcn5*	*Lgr5*	*Tmtc2*
Il18	*Maob*	*Sec16b*	*Cutal*	*Pdk2*
Gda	*Defa34*	*Akr1b7*	*Tnfrsf10b*	*Arhgef18*
Lyz1	*Slc25a20*	*Retsat*	*Sult1b1*	*Ccl25*
Aadac	*Hpd*	*Cnn2*	*Slc9a2*	*Eci1*
Entpd5	*Tmed6*	*Cbr1*	*Fcgrt*	*Fam3c*
Slc5a4b	*Gstk1*	*Cdc27*	*Cntrob*	*Hsd17b13*
Acsl5	*Acbd4*	*Ccdc57*	*Cdip1*	*Nap1l1*
Fxyd3	*Prom1*	*Polg2*	*Dnmt1*	*Vdr*
Chpt1	*Tmem139*	*Gpt*	*Akp3*	*Fah*
Pgrmc1	*Tnfrsf1b*	*Reep6*	*Gnpda1*	*Rhebl1*

Trim15	*Becn1*	*Fbxo9*	*Tspan1*	*Kyat1*
Hagh	*Duox2*	*Snrnp70*	*Ephx2*	*Abcg5*
Aqp4	*Zfyve21*	*Cyp2c55*	*Pdia5*	*Nags*
Hexb	*Ly6e*	*Agr2*	*Hsdl2*	*Cald1*
Bcl3	*Car12*	*Marchf6*	*Dgkh*	*Gdap2*
Mylk3	*Notch1*	*Acot13*	*D630003M21Rik*	*Ppa1*
Hadhb	*Tmem184a*	*Plin2*	*Bcl2l11*	*Prmt2*
Vipr1	*Nr4a1*	*Casp6*	*Snx20*	*Dhrs11*
Foxa2	*Ugt2b35*	*Rmdn1*	*Ppara*	*Slc4a5*
Sar1b	*Fam53c*	*Cyp1b1*	*Mras*	*Dhrs4*
Gstm2	*Reep5*	*Tmem236*	*Flot2*	*Acaa1a*
Lama5	*Gss*	*C1qtnf1*	*Mme*	*Maf*
Adamts15	*Ccs*	*Piwil4*	*Pola1*	*Tmcc1*
Apol9b	*Gpatch2l*	*Edn2*	*Srgap2*	*Rassf1*
Oat	*A1cf*	*Sytl3*	*Chd4*	*Dmbt1*
Nat8f5	*Cyp2b10*	*Pttg1ip*	*Impdh1*	*St3gal1*
Hsf2	*Ano7*	*Soat1*	*Frmd8*	*Rorc*
Ush1c	*Jak3*	*Tigd3*	*Tnfaip8*	*Esd*
Nfe2l1	*Dnajc13*	*Lipa*	*Adh1*	*Gpx1*
Prps2	*Fstl4*	*Dpyd*	*Axin2*	*Ago1*
Por	*Cabin1*	*Galm*	*Gpd1*	*Plbd1*
Rnf128	*Prxl2c*	*Slc51a*	*Rrbp1*	*Inf2*
Zdhhc3	*Plekhg3*	*Snn*	*4930523C07Rik*	
